# Four New Anthraquinones with Histone Deacetylase Inhibitory Activity from *Ventilago denticulata* Roots

**DOI:** 10.3390/molecules27031088

**Published:** 2022-02-06

**Authors:** Nattika Hangsamai, Kanokwan Photai, Thidathep Mahaamnart, Somdej Kanokmedhakul, Kwanjai Kanokmedhakul, Thanaset Senawong, Siripit Pitchuanchom, Mongkol Nontakitticharoen

**Affiliations:** 1 Natural Products Research Unit, Department of Chemistry and Center of Excellence for Innovation in Chemistry, Faculty of Science, Khon Kaen University, Khon Kaen 40002, Thailand; nattika.h@kkumail.com (N.H.); kanokwan_kp@kkumail.com (K.P.); m_thidathep@kkumail.com (T.M.); somdej@kku.ac.th (S.K.); kwanjai@kku.ac.th (K.K.); 2 Natural Products Research Unit, Department of Biochemistry, Faculty of Science, Khon Kaen University, Khon Kaen 40002, Thailand; sthanaset@kku.ac.th; 3 Department of Chemistry and Center of Excellence for Innovation in Chemistry, Faculty of Science, Mahasarakham University, Maha Sarakham 44150, Thailand; siripit.p@msu.ac.th

**Keywords:** *Ventilago denticulata*, anthraquinone, HDAC inhibitor, cytotoxicity

## Abstract

Chromatographic separation of the crude extracts from the roots of *Ventilago denticulata* led to the isolation of four new anthraquinones, ventilanones L–O (**1**–**4**), together with eight known anthraquinones (**5**–**12**). Their structures were elucidated by spectroscopic methods (UV, IR, ^1^H NMR, ^13^C NMR, and 2D NMR) and mass spectrometry (MS), as well as comparison of their spectroscopic data with those reported in the literature. HDACs inhibitory activity evaluation resulted that compound **2** exhibited moderate antiproliferative activity against HeLa and A549 cell lines but nontoxic to normal cell. Molecular docking indicated the phenolic functionality of **2** plays crucial interactions with class II HDAC4 enzyme.

## 1. Introduction

*Ventilago* is a genus of the plants in the family of *Rhamnaceae*, which contains around 40 species worldwide [1]. *Ventilago denticulata* Willd. (Synonym of: *Ventilago calyculata* Tul.), one of nine species found in Thailand [2], is native to Indian subcontinent to China and Indo-China region. It is a climbing shrub, spreading throughout tropical evergreen forests. The plant is locally recognized in Thai as Rang daeng (Central), Kong kaep (Northern), and Song daeng (Peninsular). Thai traditional medicine uses leaves of *V. denticulata* for the treatments of diuretics, arthritis, and hyperglycemia, whereas vines are used to treat muscle pain [3,4]. According to previous phytochemical investigations of *V. denticulata* (*V. calyculata)*, several classes of bioactive compounds have been reported including anthraquinones from vine [4], root bark [5,6], and root [7]; naphthalene derivatives from vine [4]; benzisochromanquinones from vine [4], root [7], and trunk [8]; and flavonoids from the trunk [8]. These natural products demonstrated their biological principles such as antioxidant, cytotoxic, antibacterial, antifungal, and phosphodiesterase inhibitory activities [4,8]. It is obvious that *V. denticulata* is rich in pharmaceutical active compounds related to the polyketide biosynthetic pathway. As part of our ongoing search for potent anticancer agents from plants, we are interested in anthraquinones, one of the polyketide-derived secondary metabolites from *V. denticulata*, due to their promising anticancer activity [9,10,11]. Anthraquinones have a planarity of 9,10-dioxoanthracene core structure, which can embed into active site pocket of targeting enzymes, resulting enzyme suppression, arresting cell cycle, and inducing cell apoptosis. To broaden the study of new naturally occurring anthraquinones and their anticancer potency, we report herein the isolation and characterization of anthraquinones from the roots of *V. denticulata* and evaluate their histone deacetylase (HDAC) inhibitory activity.

## 2. Results and Discussion

### 2.1. Structure Characterization

**Figure 1:**
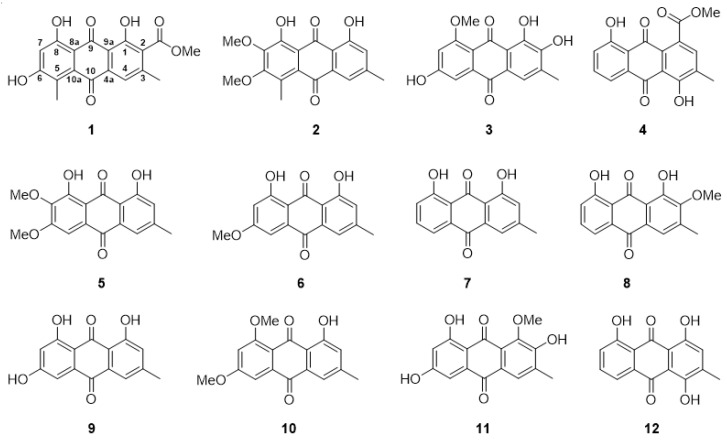
Structures of the isolated anthraquinones (**1**–**12**) from the roots of *V. denticulata*.

Chromatographic separation of anthraquinones from the roots of *V. denticulata* led to the isolation of twelve anthraquinones including four new (**1**–**4**) and eight known (**5**–**12**) compounds. Their structures were elucidated by spectroscopic methods (UV, IR, ^1^H NMR, ^13^C NMR, and 2D NMR) and mass spectrometry (MS), as well as comparison of their spectroscopic data with those reported in the literature (Figure 1 and Supplementary Figures S1–S48). The known isolated anthraquinones were characterized as 7-methoxyphyscion (**5**) [12]—which was isolated as a naturally occurring anthraquinone for the first time—physcion (**6**) [13], chrysophanol (**7**) [14], 2-methoxychrysophanol (**8**) [15], emodin (**9**) [16], emodin-6,8-dimethyl ether (**10**) [17], 2-hydroxyemodin-1-methyl ether (**11**) [18], and islandicin (**12**) [19]. It should be noted that compound **8** was isolated from the genus *Ventilago* for the first time, whereas compound **11** was found for the first time from *V. denticulata*. However, compounds **6**–**7**, **9**–**10**, and **12** were previously isolated from the root barks of *V. calyculata.*

**Figure 2:**
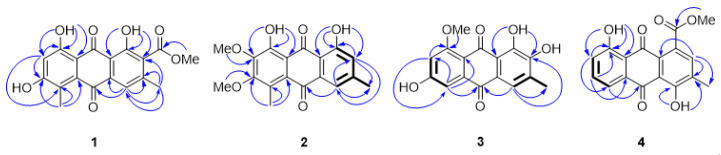
The key HMBC (arrow line) and COSY (bold line) correlations of compounds **1**–**4**.

Compound **1** was obtained as a red-brown solid. Its molecular formula, C_18_H_14_O_7_, was established by a [M + Na]^+^ ion peak at *m*/*z* 365.0638 (calcd for C_18_H_14_NaO_7_ 365.0632) in the HRESITOFMS, indicating twelve indices of hydrogen deficiency. The UV spectrum showed maximal absorptions at 220, 255, 291, and 499 nm. The IR spectrum displayed absorption bands of hydroxyl (3232 cm^−1^), carbonyl (1731 cm^−1^), quinone carbonyl (1672 and 1620 cm^−1^), and aromatic (1604 cm^−1^) functionalities. The ^1^H and ^13^C NMR data (Table 1 and Table 2, respectively) as well as the spectroscopic data mentioned above suggested **1** was a 1,8-dihydroxyanthraquinone derivative. Two chelated hydroxyl protons appeared as two singlets at δ_H_ 12.28 (1H, s, 1-OH) and 12.59 (1H, s, 8-OH). Two anthraquinone carbonyl carbons resonated at δ_C_ 189.4 (C-9) and 183.4 (C-10). The HMBC data (Figure 2) supported the connections of 1-OH and 8-OH to the oxygenated aromatic carbons C-1 (δ_C_ 157.3) and C-8 (δ_C_ 163.2), respectively, by the correlations of 1-OH to C-1, C-2 (δ_C_ 127.6), and C-9a (δ_C_ 113.7) and 8-OH to C-7 (δ_C_ 106.8), C-8, and C-8a (δ_C_ 109.1). The methoxy group at δ_H_ 3.89 (3H, s)/δ_C_ 52.6 correlated to the conjugated carbonyl carbon (δ_C_ 166.2) revealed the presence of methyl ester moiety, which was attached to C-2. This attachment was found in well agreement with the anthraquinone biosynthesis [20]. A methyl groups appeared at δ_H_ 2.36 (3H, s, 3-CH_3_)/*δ*_C_ 19.7 was connected to C-3 (δ_C_ 144.2), supported by the HMBC correlations of 3-CH_3_ to C-2, C-3, and an aromatic methine carbon C-4 (δ_C_ 120.4). Another aromatic methyl group showed resonance signal at δ_H_ 2.45 (3H, s, 5-CH_3_)/δ_C_ 13.0, which was located at C-5 (*δ*_C_ 124.7), confirmed by the correlations of 5-CH_3_ to C-5, an oxygenated aromatic carbon C-6 (δ_C_ 165.2), and C-10a (δ_C_ 131.5). An aromatic proton appeared singlet at δ_H_ 7.52 (1H, s) belonged to H-4, which was confirmed by HMBC data; H-4 to C-3, CH_3_-3, C-4a (δ_C_ 134.5), and C-10. The higher field aromatic methine proton H-7 signaled singlet at δ_H_ 6.68 (1H, s)/δ_C_ 106.8, confirmed by the HMBC correlations of H-7 to C-5, C-6, C-8, and C-8a. Based on the spectroscopic data above, **1** was identified as a new 1,8-dihydroxyanthraquinone derivative, namely ventilanone L.

Compound **2** was obtained as an orange solid. Its molecular formula, C_18_H_16_O_6_, was established by a [M − H]^−^ ion peak at *m/z* 327.0876 (calcd for C_18_H_15_O_6_ 327.0896) in the HRESITOFMS, indicating eleven indices of hydrogen deficiency. The UV spectrum showed maximal absorptions at 208, 225, 264 and 438 nm. The IR spectrum exhibited absorption bands of hydroxyl (3476 cm^−1^), quinone carbonyl (1625 cm^−1^), and aromatic (1460 cm^−1^) functionalities. The ^1^H and ^13^C NMR data of **2** (see Table 1 and Table 2), suggesting the presence of a 1,8-dihydroxyanthraquinone skeleton. Two chelated hydroxyl protons appeared at δ_H_ 11.91 (1H, s, 1-OH) and 13.03 (1H, s, 8-OH), which were attached to C-1 (δ_C_ 162.2) and C-8 (δ_C_ 157.0), respectively, confirmed by the HMBC correlations (Figure 2) of 1-OH to C-1, C-2 (δ_C_ 123.4), and C-9a (δ_C_ 113.7) and 8-OH to C-7 (δ_C_ 145.7), C-8, and C-8a (δ_C_ 113.6). Two anthraquinone carbonyl carbons showed resonance signals at δ_C_ 192.1 (C-9) and 183.8 (C-10). Two aromatic methyl groups were found in **2**, as same as in **1**. The methyl group appeared at δ_H_ 2.59 (3H, s, 5-CH_3_)/δ_C_ 14.2 was introduced to C-5 (δ_C_ 126.8), based on the HMBC correlations of 5-CH_3_ to C-5, C-6 (δ_C_ 158.5), and C-10a (δ_C_ 131.0). An aromatic methyl group resonated doublet of doublet at δ_H_ 2.42 (3H, dd, *J* = 0.7, 0.7 Hz, 3-CH_3_)/δ_C_ 22.5, indicating this methyl group was between two aromatic methine protons H-2 (δ_H_ 7.01 (1H, dd, *J* = 1.7, 0.7 Hz)) and H-4 (δ_H_ 7.54 (1H, dd, *J* = 1.7, 0.7 Hz, H-4)), confirmed by the COSY correlations of an allylic proton-proton couplings (Figure 2). The aromatic proton H-2 attached to C-2 (*δ*_C_ 123.4), supported by the correlations of H-2 to C-1, CH_3_-3, C-4 (δ_C_ 158.5), C-9a, and C-10 in the HMBC data. The COSY spectrum displaying a meta-coupled between H-2 and H-4 with the coupling constant value of 1.7 Hz also provided structure authentically. Position of the aromatic proton H-4 also confirmed by the HMBC correlations of H-4 to C-2, CH_3_-3, and C-10. Two aromatic methoxy groups appeared at δ_H_ 3.97 (3H, s, 6-OCH_3_)/δ_C_ 61.2 and δ_H_ 4.01 (3H, s, 7-OCH_3_)/δ_C_ 61.4 connected to aromatic oxygenated carbons C-6 and C-7, respectively, deduced from the HMBC correlations of 6-OCH_3_ to C-6 and 7-OCH_3_ to C-7. Based on the spectroscopic data above, **2** was identified as a new 1,8-dihydroxyanthraquinone derivative, namely ventilanone M.

Compound **3** was obtained as an orange-brown solid. Its molecular formula, C_16_H_12_O_6_, was established by a [M + H]^+^ ion peak at *m/z* 301.0707 (calcd for C_16_H_13_O_6_ 301.0690) in the HRESITOFMS, indicating ten indices of hydrogen deficiency. The UV spectrum showed maximal absorptions at 229, 281, 308, and 427 nm. The IR spectrum showed absorption bands of hydroxyl (3459 cm^−1^), quinone carbonyl (1617 cm^−1^), and aromatic (1560 cm^−1^) functionalities. The ^1^H and ^13^C NMR data of **3** (see Table 1 and Table 2), as well as the data mentioned above, suggested the presence of anthraquinone structure. Two characteristics anthraquinone carbonyl carbons resonated at δ_C_ 186.9 (C-9) and 180.8 (C-10). An aromatic methine proton H-4 appeared doublet at δ_H_ 7.47 (1H, d, *J* = 0.6 Hz)/δ_C_ 121.4. Comparing to those of compounds **1** and **2**, this proton nuclei resonated at lower frequency due to shielding effect from hydroxyl group at C-2 (δ_C_ 150.6). The structure of **3** was supported by the COSY correlations of an allylic proton-proton couplings between H-4 and 3-CH_3_ (δ_H_ 2.25 (3H, d, *J* = 0.6 Hz)/δ_C_ 16.3) (Figure 2) and the HMBC correlations of H-4 to C-2, CH_3_-3, and C-10 (Figure 2). A chelated hydroxyl proton observed at δ_H_ 13.65 (1H, s) was assigned to be 1-OH, based on the HMBC correlations of 1-OH to C-2 and C-9a (δ_C_ 115.0). An aromatic proton with resonance signals at δ_H_ 7.22 (1H, d, *J* = 2.4 Hz)/δ_C_ 106.9 was determined to be H-5, which showed HMBC correlations to C-7 (δ_C_ 104.3), C-8a (δ_C_ 112.7), and C-10. An aromatic proton H-7 appeared high field at δ_H_ 6.81 (1H, d, *J* = 2.4 Hz)/δ_C_ 104.3, as a resulted of its ortho-position to two oxygenated groups (6-OH and 8-OCH_3_). The assignment of H-7 was also confirmed by an aromatic meta-coupled (2.4 Hz) between H-5 and H-7 and the HMBC correlations of H-7 to C-5, C-8 (δ_C_ 164.6), and C-8a. A methoxy group appeared at δ_H_ 3.92 (3H, s, 8-OCH_3_)/δ_C_ 56.3 was introduced to C-8, based on the HMBC correlation of 8-OCH_3_ to C-8. Interestingly, the structure of **3** was almost identical to 2-hydroxyemodin 1-methyl ether (**11**), an anthraquinone isolated from *V. leiocarpa* [18], except the aromatic methoxy group of that anthraquinone located at C-1. According to the ^1^H NMR data, it was unambiguous that structure of **3** completely distinguished from **11**. The aromatic proton H-4 of **3**, which was para-positioned to the chelated hydroxyl group 1-OH, resonated more lower field (7.49 ppm) than **11**′s (7.88 ppm). Based on the spectroscopic data above, **3** was identified as a new anthraquinone derivative, namely ventilanone N.

Compound **4** was obtained as an orange solid. Its molecular formula, C_16_H_12_O_6_, was established by a [M − H]^−^ ion peak at *m/z* 311.0572 (calcd for C_17_H_11_O_6_ 311.0561) in the HRESITOFMS, indicating twelve indices of hydrogen deficiency. The UV spectrum showed maximal absorptions at 226, 255, 287 and 429 nm. The IR spectrum displayed absorption bands of hydroxyl (3462 cm^−1^), carbonyl (1731 cm^−1^), quinone carbonyl (1620 cm^−1^), and aromatic (1468 cm^−1^) functionalities. The ^1^H and ^13^C NMR data of **4** (see Table 1 and Table 2), as well as the data mentioned above suggested **4** was an anthraquinone derivative. Two anthraquinone carbonyl carbons observed resonance signals at δ_C_ 192.5 (C-9) and 181.6 (C-10). Two chelated hydroxyl protons signaled at δ_H_ 12.34 (1H, s) and 11.94 (1H,s) were corresponding to 4-OH and 8-OH, respectively, confirmed by the HMBC correlations (Figure 2) of 4-OH to C-3 (δ_C_ 129.2), C-4 (δ_C_ 159.9), and C-9a (δ_C_ 114.3) and 8-OH to C-7 (δ_C_ 125.1), C-8 (δ_C_ 162.8), and C-8a (δ_C_ 115.9). A singlet aromatic proton signaled at δ_H_ 7.66 (1H, s)/δ_C_ 121.7, dedicated to H-2 which showed correlations to C-1 (δ_C_ 133.7), C-3, and C-9a, based on the HMBC data. An aromatic methyl group at δ_H_ 2.44 (3H, s, 3-CH_3_)/δ_C_ 20.7 exhibited the HMBC correlations to C-2, C-3, and C-4a (δ_C_ 146.6). Three aromatic methine protons appeared at δ_H_ 7.81 (1H, dd, *J* = 7.5, 1.1 Hz, H-5), 7.67 (1H, dd, *J* = 8.2, 7.5 Hz, H-6), and 7.29 (1H, dd, *J* = 8.2, 1.1 Hz, H-7) were corresponding to aromatic methine carbons C-5 (δ_C_ 120.4), C-6 (δ_C_ 137.6), and C-7 (δ_C_ 125.1), respectively. The assignments confirmed by the COSY correlations (Figure 2) of the ortho- and meta-couples between these three protons, which deduced from their coupling constants. The HMBC spectrum showed the correlations of H-5 to C-7, C-8a, and C-10, H-6 to C-8, C-10, and C-10a (δ_C_ 133.6), H-7 to C-5, and C-8a. The presence of a methyl ester group, which connected to C-1 was confirmed by the methoxy group signaled at δ_H_ 3.98 (3H, s)/δ_C_ 53.3 connected to a conjugated ester carbon appeared at *δ*_C_ 166.7 (1-COO) confirmed by the HMBC data. Based on the spectroscopic data above, **4** was identified as a new anthraquinone derivative, namely ventilanone O.

### 2.2. HDAC Inhibitory Activity

Anthraquinones **2**, **6**, and **9** were selected for screening by the Fluor-de-Lyse™ in vitro fluorescence activity assay kit as measuring total HDAC inhibitory activity in HeLa nuclear extract (Table 3). Among the tested compounds, **2** showed the highest percentage of HDAC inhibition value (61.27%), whereas **6** and **9** demonstrated HDAC inhibition lower than 50%.

### 2.3. Physicochemical Properties

Drug-likeness is a useful concept in drug design that increases the chances of chemical entities and avoids drug development failure. In this study, the SwissADME web server (http://swissadme.ch, accessed on 30 December 2021) was performed to estimate the physicochemical features of anthraquinones **1**–**4**, to assess their drug-likeness [21]. The results (Table 4) showed that **1**–**4** presented no violation of Lipinski’s rules. The molecular weights, number of hydrogen-bond acceptors, and number of hydrogen-bond donors were within the accepted values of less than 500, 10, and 5, respectively. Their LogP values were within the range of 2.60 to 3.65. Additionally, **2** showed the lowest topological polar surface area (TPSA), indicating the most favorable drug-likeness.

### 2.4. Molecular Docking Study

To predict the possibility of being HDAC isoform-selective inhibitor, **2** was docked into the catalytic pockets of the representative isoform of class I (HDAC1, HDAC2, and HDAC8) and class II (HDAC4 and HDAC7). The available crystal structures of HDAC1, HDAC2, HDAC4, HDAC7, and HDAC8 were obtained from the Protein Data Bank (https://www.rcsb.org, accessed on 30 December 2021). The docking results are summarized in Table 5. For HDAC4 and HDAC7 templates, **2** had a low binding energy, which has significance for HDAC class II. The docking studies of **2** with HDAC4 revealed that it has the lowest binding energy of −6.85 kcal/mol. It is also against HDAC4 and HDAC7 with Ki values of 9.49 and 5.29 μM, respectively.

The pharmacophore model of HDAC inhibitors consists of three main parts: a zinc-binding group (ZBG), a linker, and a hydrophobic cap (CAP). The role of ZBG is to bind Zn^2^^+^ cofactor at enzyme active site that directly inhibits enzymatic activity. The linker domain resembles the substrate and is able to bind the cylindrical pocket of the HDAC active site. Meanwhile, CAP interacts with the surface and closes the cylindrical pocket of the active site. In HDAC4, a Zn^2^^+^ cofactor binds to charge-relay system consisting of two aspartate residues (Asp196 and Asp290) and one histidine residue (His198) [22].

**Figure 3:**
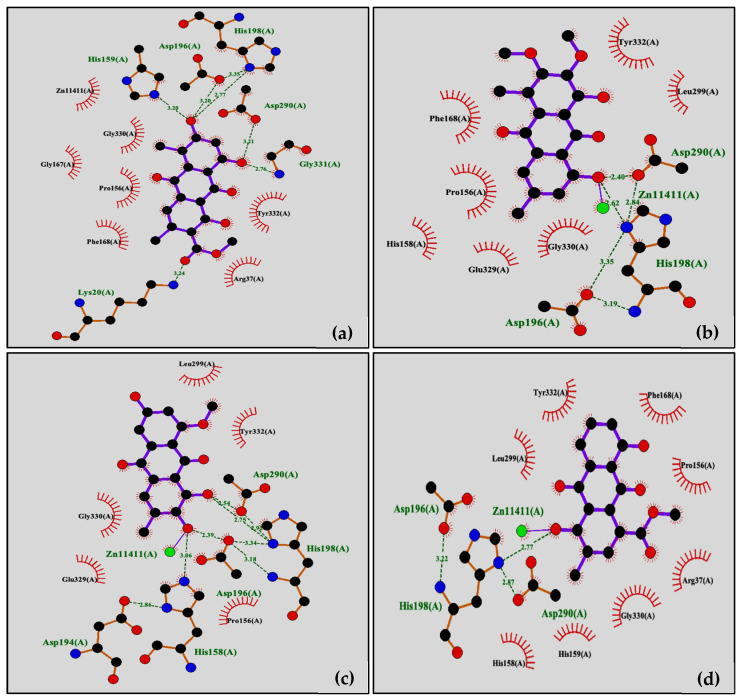
Binding interactions of the isolated compounds docked into HDAC4 template with hydrogen-bonds (dash bond): (**a**) compound **1**; (**b**) compound **2**; (**c**) compound **3**; (**d**) compound **4**.

The binding modes and interactions of anthraquinones **1**–**4** with HDAC4 template were studied in order to obtain more insights into their HDAC inhibitory activity (Figure 3). Compound **1** formed six hydrogen bonds with His159, His198, Asp196, Asp290, Gly331, and Lys20, whereas no interaction with catalytic zinc ion (Figure 3a). Docking mode of 2 shows two hydrogen bonds of the hydroxyl group at C-1 with *N*-imidazole ring of His198 and Asp290, as the charge-relay system in HDAC4 catalytic site. In addition, this hydroxyl group also approached the zinc ion to establish ionic interaction (Figure 3b). According to the result, the phenolic component of **2** interacts with HDAC4 enzyme through dual binding mode, including CAP and ZBG domains. Although the hydroxyl group at C-1 of **3** interacted with His198 and Asp290, as found in **2**, compound **3** showed no hydrophobic interaction with Phe168 and Pro156 residues (Figure 3c). Interestingly, **4** was found to have a hydrogen-bond with His198, together with hydrophobic and ZBG interactions (Figure 3d).

### 2.5. MTT Assay

The MTT assay of **2** was carried out to gain more details regarding the anticancer activity of the potent HDAC inhibitor. To complete the evaluation of this potent HDAC inhibitor, the antiproliferative activity was determined in human cervical cancer (HeLa), human lung cancer (A549), and human breast adenocarcinoma cancer (MCF-7) cells. Moreover, **2** was also determined in noncancer cells (Vero cells) and cisplatin was used as drug control (Table 6). Results indicated that **2** possessed potent capacity against HeLa and A549 cell lines for 72 h with IC_50_ values of 160.87, and 177.32 μM, respectively. However, **2** showed less active against MCF-7 cell line and appeared less toxic to normal cells.

## 3. Materials and Methods

### 3.1. General Experimental Procedures

Melting points were determined on a SANYO MPU250BM3.5 melting point apparatus and were uncorrected. UV spectra were recorded on an Agilent 8453 UV-visible spectrophotometer (Agilent Technologies, Santa Clara, CA, USA). IR spectra were determined by a Bruker Tensor 27 FT-IR spectrophotometer (Bruker, Ettlingen, Germany). The ^1^H and ^13^C NMR spectra were obtained from a Bruker AVANCE NEO (400 MHz) spectrometer (Bruker, Rheinstetten, Germany). Chemical shifts were reported on the *δ* (ppm) scale using chloroform-*d*_1_, methanol-*d*_4_, acetone-*d*_6_, and DMSO-*d*_6_ as the solvent and residual of those solvents as internal standards. HRESITOFMS were recorded on a Finnigan Mat INCOS 50 and Micromass LTC mass spectrometers and a Finnigan MAT-90, a microTOF, Bruker Daltonics, and a Finnigan LC-Q mass spectrometer. Silica gel 60 (Merck, Darmstadt, Germany) 0.063–0.200 mm or less than 0.063 mm and Sephadex LH-20 (Amersham Pharmacia, Biotech AB, Sweden) were used for column chromatography. Preparative thin layer chromatography was carried out on glass supported silica gel plates using silica gel 60 GF254 for thin-layer chromatography (Merck, Darmstadt, Germany). Thin layer chromatography was performed on precoated silica gel 60 PF254, aluminum sheets (Merck, Darmstadt, Germany).

### 3.2. Plant Materials

The roots of *V. denticulata* were collected from local conservation forest in Lampang province, Thailand, in November 2017. The plant was identified by Surapon Saensouk, Mahasarakham University, Thailand. The voucher specimen (no. SPMSU002) was deposited at Mahasarakham University Herbarium, Thailand.

### 3.3. Extraction and Isolation

The air-dried powdered roots of *V. denticulata* (14 kg) were ground and extracted successively with organic solvents including hexane, dichloromethane (CH_2_Cl_2_), ethyl acetate (EtOAc), and methanol (MeOH) at room temperature. Removal of solvents from the extracts under reduced pressure afforded crude hexane (110.0 g), CH_2_Cl_2_ (417.0 g), EtOAc (150.0 g), and MeOH (749.0 g) extracts, respectively.

The hexane extract (108.0 g) was separated by column chromatography (CC) using silica gel, eluted by gradient system of hexane:EtOAc and EtOAc:MeOH to give nine fractions, RH1-RH9. Fraction RH5 (12.8 g) was further separated over silica gel flash column chromatography (FCC), eluted by gradient system of hexane:EtOAc and EtOAc:MeOH to afford nine fractions, RH5.1-RH5.9. Fraction RH5.5 (0.780 g) was then chromatographed over silica gel CC, eluted by gradient system of hexane:EtOAc and EtOAc:MeOH to obtain compound **8** (101.5 mg) and ten fractions, RH5.5.1–RH5.5.10. Fraction RH5.5.4 (0.800 g) was then separated using silica gel CC, eluted with gradient system of hexane:EtOAc and EtOAc:MeOH to afford ten fractions, RH5.5.4.1–RH5.5.4.10. Fraction RH5.5.4.10 (10.5 mg) was purified by preparative thin layer chromatography (prep. TLC), eluted with an isocratic system of hexane:acetone (80:20) to afford compound **5** (4.9 mg). Fraction RH8 (7.2 g) was separated by silica gel CC, eluted by gradient system of hexane:EtOAc and EtOAc:MeOH to give seven fractions, RH8.1–RH8.7. Fraction RVDH8.2 (5.8 g) was separated by silica gel CC, eluted by gradient system of hexane:EtOAc and EtOAc:MeOH to give seven fractions, RH8.2.1–RH8.2.7. Fraction RH8.2.3 (0.925 g) was further separated over silica gel CC, eluted by gradient system of hexane:EtOAc and EtOAc:MeOH to give compound **10** (8.8 mg).

The CH_2_Cl_2_ extract (150.0 g) was subjected to silica gel FCC, eluted with gradient system of hexane:CH_2_Cl_2_, CH_2_Cl_2_:EtOAc and EtOAc:MeOH to give six fractions, RC1–RC6. Fraction RC2 (13.8 g) was further separated by silica gel CC, eluted with gradient system of hexane:EtOAc and EtOAc:MeOH to give nine fractions, RC2.1–RC2.9. The precipitate in fraction RC2.2 was filtered out to give compound **2** (5.7 mg). Fraction RC2.3 (0.575 g) was further separated by Sephadex LH-20 CC, eluted with MeOH to give four fractions, RC2.3S1–RC2.3S4. Fraction RC2.3S3 (140.0 mg) was purified by silica gel CC, eluted with gradient system of hexane:EtOAc to give six fractions, RC2.3S3.1–RC2.3S3.6. The precipitate in fraction RC2.3S3.1 was filtered out to give compound **6** (4.4 mg). Fraction RC2.3S3.5 (42.5 mg) was subjected to Sephadex LH-20 CC, using MeOH as eluent, afforded compound **4** (3.5 mg). Fraction RC5 (3.0 g) was subjected to silica gel CC, eluted with gradient system of hexane:EtOAc and EtOAc:MeOH to give seven fractions, RC5.1–RC5.7. Fraction RC5.3 (12.5 mg) was purified by Sephadex LH-20 CC, eluted with MeOH to yield compound **1** (3.0 mg). Fraction RC5.4 (1.105 g) was separated by silica gel CC, eluted with gradient system of hexane:EtOAc to give compound **9** (17.2 mg).

The EtOAc extract (130.0 g) was subjected to silica gel FCC, eluted with gradient system of hexane:EtOAc and EtOAc:MeOH to give nine fractions, RE1–RE9. Fraction RE3 (4.0 g) was separated over silica gel CC, eluted with gradient system of CH_2_Cl_2_:EtOAc and EtOAc:MeOH to give eight fractions, RE3.1–RE3.8. Fraction RE3.2 (91.0 mg) was purified using prep. TLC, eluted with an isocratic system of hexane:acetone (80:20) to afford compounds **7** (4.8 mg) and **12** (2.3 mg). Fraction RE5 (2.2 g) was separated by silica gel CC, eluted with gradient system of CH_2_Cl_2_:EtOAc and EtOAc:MeOH to yield eight fractions, RE5.1–RE5.8. Fraction RE5.7 (0.690 g) was further purified silica gel CC, eluted with gradient system of CH_2_Cl_2_:EtOAc and EtOAc:MeOH to obtain compound **11** (5.2 mg). Fraction RE7 (8.0 g) was separated by silica gel CC, eluted with gradient system of hexane:EtOAc and EtOAc:MeOH to afford seven fractions, RE7.1–RE7.7. The solid in fraction RE7.5 was filtered out to obtain compound **3** (5.6 mg).

Ventilanone L (**1**): Red–brown solid; mp 268.4–269.5 °C (MeOH); UV (MeOH) λ_max_ (log ɛ): 220 (3.49), 255 (3.85), 291 (3.26), 499 (3.07) nm; IR (ATR) *ν*_max_: 3232, 2955, 2923, 2851, 1731, 1620, 1391, 1354, 1235 cm^−1^; HRESITOFMS: [M + Na]^+^ *m/z* 365.0638 (calcd for C_18_H_14_NaO_7_ 365.0632); ^1^H and ^13^C NMR data, see Table 1 and Table 2.

Ventilanone M (**2**): Orange solid; mp 153.8–154.2 °C (CH_2_Cl_2_); UV (MeOH) λ_max_ (log ɛ): 208 (4.31), 225 (4.29), 264 (4.24), 438 (3.93) nm; IR (ATR) *ν*_max_: 2943, 2851, 1625, 1460, 1405, 1379 cm^−1^; HRESITOFMS: [M − H]^−^ *m/z* 327.0876 (calcd for C_18_H_15_O_6_ 327.0869); ^1^H and ^13^C NMR data, see Table 1 and Table 2.

Ventilanone N (**3**): Orange-brown solid; mp (decomp) 290.5 °C (EtOAc); UV (MeOH) λ_max_ (log ε): 229 (4.24), 281 (4.32), 308 (3.90), 427 (3.84) nm; IR (ATR) *ν*_max_: 3459, 3091, 2965, 1617, 1560, 1430, 1340 cm^−1^; HRESITOFMS: [M + H]^+^ *m/z* 301.0707 (calcd for C_16_H_13_O_6_ 301.0690); ^1^H and ^13^C NMR data, see Table 1 and Table 2.

Ventilanone O (**4**): Orange solid; mp 174.8–175.2 °C (CH_2_Cl_2_); UV (MeOH) λ_max_ (log ɛ): 226 (4.12), 255 (3.86), 287 (3.58), 429 (3.58) nm; IR (ATR) *ν*_max_: 3055, 2955, 2853, 1723, 1673, 1622, 1598, 1496, 1450, 1374 cm^−1^. HRESITOFMS: [M − H]^−^ (calcd for C_17_H_11_O_6_ 311.0561); ^1^H and ^13^C NMR data, see Table 1 and Table 2.

7-methoxyphyscion (**5**): Yellow solid; mp 192.8–193.4 °C (CH_2_Cl_2_); UV (MeOH) λ_max_ (log ɛ): 217 (4.33), 277 (4.30), 431 (3.92) nm; IR (ATR) *ν*_max_: 3384, 2922, 2851, 1671, 1624, 1604, 1560, 1478, 1450, 1407, 1364, 1333, 1306, 1265 cm^−^^1^; HRESITOFMS *m/z* 337.0692 [M + Na]^+^ (calcd for C_17_H_14_NaO_6_ 337.0683); ^1^H NMR data (400 MHz, CDCl_3_) δ 12.25 (1H, s, OH-8), 12.01 (1H, s, OH-1) 7.61 (1H, s, H-4), 7.44 (1H, s, H-5), 7.06 (1H, s, H-2), 4.04 (3H, s, OCH_3_-6), 4.01 (3H, s, OCH_3_-7), 2.45 (3H, s, CH_3_-3); ^13^C NMR data (100 MHz, CDCl_3_) δ 191.7 (C-9), 181.7 (C-10), 162.6 (C-1), 158.7 (C-6), 156.6 (C-8), 148.9 (C-3), 141.8 (C-7), 133.2 (C-4a), 129.5 (C-10a), 124.5 (C-2), 121.5 (C-4), 113.8 (C-9a), 112.0 (C-8a), 104.7 (C-5), 61.2 (OCH_3_-7), 56.7 (OCH_3_-6), 22.3 (CH_3_-3).

Physcion (**6**): Yellow solid; mp 198.5–199.8 °C (CH_2_Cl_2_); UV (MeOH) λ_max_ (log ε): 224 (3.78), 264 (3.54), 287 (3.52), 434 (3.35) nm; IR (KBr) *ν*_max_: 2959, 2923, 2825, 1735, 1480, 1366, 1259, 1220, 1157 cm^−^^1^; HRESIMS *m/z* 283.0617 [M − H]^−^ (calcd for C_16_H_11_O_5_ 283.0612); ^1^H NMR data (400 MHz, CDCl_3_) δ 12.31 (1H, s, OH-1), 12.11 (1H, s, OH-8), 7.62 (1H, s, H-4), 7.36 (1H, s, H-5), 7.08 (1H, s, H-2), 6.68 (1H, s, H-7), 3.94 (3H, s, OCH_3_-6), 2.45 (3H, s, CH_3_-3); ^13^C NMR data (100 MHz, CDCl_3_) δ 191.0 (C-9), 182.2 (C-10), 166.7 (C-6), 165.3 (C-8), 162.7 (C-1), 148.6 (C-3), 135.4 (C-10a), 133.4 (C-4a), 124.7 (C-2), 121.4 (C-4), 113.8 (C-9a), 110.4 (C-8a), 108.4 (C-5), 106.9 (C-7), 56.2 (OCH_3_-6), 22.3 (CH_3_-3).

Chrysophanol (**7**): Orange-red solid; mp 195.5–196.7 °C (CH_2_Cl_2_); UV (MeOH) λ_max_ (log ε): 225 (4.22), 256 (3.97), 287 (3.70), 429 (3.67) nm; IR (ATR) ν_max_: 3005, 2963, 2924, 2854, 1676, 1626, 1567, 1452, 1397 cm^−^^1^; HRESITOFMS m/z: 253.0603 [M − H]ˉ (calcd for C_15_H_9_O_4_ 253.0506); ^1^H NMR data (400 MHz, CDCl_3_) δ 12.13 (1H, s, OH-8), 12.02 (1H, s, OH-1), 7.82 (1H, dd, *J* = 6.6, 0.9 Hz, H-5), 7.66 (1H, t, *J* = 8.2 Hz, H-6), 7.65 (1H, brs, H-4), 7.29 (1H, dd, *J* = 7.4, 0.9 Hz, H-7), 7.10 (1H, s, H-2), 2.47 (3H, s, CH_3_-3); ^13^C NMR (100 MHz, CDCl_3_) δ 192.7 (C-9), 182.2 (C-10), 162.9 (C-8), 162.6 (C-1), 149.5 (C-3), 137.1 (C-6), 133.8 (C-4a), 133.4 (C-10a), 124.7 (C-7), 124.5 (C-2), 121.5 (C-4), 120.1 (C-5), 116.0 (C-8a), 113.9 (C-9a), 22.4 (CH_3_-3).

2-Methoxychrysophanol (**8**): Yellow solid; ^1^H NMR data (400 MHz, CDCl_3_) δ 12.37 (1H, s, OH-1), 11.97 (1H, s, OH-8), 7.83 (1H, dd, *J* =1.1, 7.5 Hz, H-5), 7.70 (1H, d, *J* =8.4 Hz, H-6), 7.69 (1H, s, H-4), 7.31 (1H, dd, *J* =1.1, 8.4 Hz, H-7), 4.00 (3H, s, OCH_3_-2), 2.46 (3H, s, CH_3_-3); ^13^C NMR data (100 MHz, CDCl_3_) δ 192.5 (C-9), 181.5 (C-10), 166.6 (C-2), 162.7 (C-8), 159.8 (C-1), 146.5 (C-3), 137.5 (C-6), 133.6 (C-10a), 129.1 (C-4a), 125.1 (C-7), 121.7 (C-4), 120.4 (C-5), 115.9 (C-8a), 114.2 (C-9a), 52.9 (OCH_3_-2), 20.7 (CH_3_-3).

Emodin (**9**): Orange solid; mp 256.9–258.7 °C (MeOH); UV (MeOH) λ_max_ (log ε): 221 (4.55), 253 (4.30), 289 (4.32), 437 (4.06) nm; IR (ATR) *ν*_max_: 3376, 2958, 2922, 2852, 1617, 1478, 1412, 1367, 1329, 1275, 1262 cm^−^^1^; HRESITOFMS *m/z* 269.0464 [M − H]ˉ (calcd for C_15_H_9_O_5_ 269.0455); ^1^H NMR data (400 MHz, CDCl_3_ + CD_3_OD) δ 7.62 (1H, d, *J* = 1.0 Hz, H-4), 7.28 (1H, d, *J* = 2.4 Hz, H-5), 7.14 (1H, d, *J* = 1.0 Hz, H-2), 6.67 (1H, d, *J* = 2.4 Hz, H-7), 2.54 (3H, s, CH_3_-3); ^13^C NMR data (100 MHz, CDCl_3_ + CD_3_OD) δ 191.2 (C-9), 183.0 (C-10), 166.5 (C-8), 165.9 (C-6), 162.9 (C-1), 149.0 (C-3), 136.1 (C-4a), 134.0 (C-10a), 124.9 (C-2), 121.6 (C-4), 114.4 (C-9a), 110.1 (C-8a), 110.0 (C-7), 108.9 (C-5), 22.2 (CH_3_-3).

Emodin 6,8-dimethyl ether (**10**): Orange-red solid, mp 213.5–214.7 °C (CH_2_Cl_2_); UV (MeOH) λ_max_ (log ε): 224 (4.71), 270 (4.44), 279 (4.45), 421 (4.11) nm; IR (ATR) *ν*_max_: 2941, 1665. 1589, 1551, 1489, 1452, 1363, 1316 cm^−^^1^; ^1^H NMR data (400 MHz, CDCl_3_) δ 13.08 (1H, s, OH-1), 7.55 (1H, d, *J* = 1.0 Hz, H-4), 7.44 (1H, d, *J* = 2.4 Hz, H-5), 7.06 (1H, d, *J* = 1.0 Hz, H-2), 6.77 (1H, d, *J* = 2.4 Hz, H-7), 4.02 (3H, s, OCH_3_-6), 3.98 (3H, s, OCH_3_-8), 2.42 (3H, s, CH_3_-3); ^13^C NMR (100 MHz, CDCl_3_) δ 187.4 (C-9), 182.9 (C-10), 165.2 (C-8), 162.9 (C-6), 162.6 (C-1), 146.9 (C-3), 137.6 (C-10a), 132.3 (C-4a), 124.8 (C-2), 120.0 (C-4), 115.2 (C-8a), 114.7 (C-9a), 104.7 (C-7), 103.9 (C-5), 56.6 (OCH_3_-6), 56.0 (OCH_3_-8), 21.9 (CH_3_-3).

2-Hydroxyemodin 1-methyl ether (**11**): Yellow solid; mp (decomp) 255.0 °C; UV (MeOH) λ_max_ (log ε): 228 (4.36), 285 (4.47), 310 (4.05), 394 (3.92) nm; IR (ATR) *ν*_max_: 3500, 1624, 1578, 1461, 1369, 1258 cm^−^^1^; ^1^H NMR data (400 MHz, CD_3_COCD_3_) δ 13.18 (1H, s, OH-8), 9.93 (1H, brs, OH-6), 9.23 (1H, brs, OH-2), 7.88 (1H, s, H-4), 7.20 (1H, d, *J* = 2.5 Hz, H-5), 6.64 (1H, d, *J* = 2.5 Hz, H-7), 3.93 (3H, s, OCH_3_-1), 2.37 (3H, s, CH_3_-3); ^13^C NMR data (100 MHz, CD_3_COCD_3_) δ 188.0 (C-9), 181.9 (C-10), 166.4 (C-8), 165.4 (C-6), 156.4 (C-2), 148.1(C-1), 136.1 (C-10a), 132.6 (C-3), 127.1 (C-4), 126.8 (C-4a), 124.8 (C-9a), 111.9 (C-8a), 108.6 (C-7), 108.1 (C-5), 62.1 (OCH_3_-1), 16.6 (CH_3_-3).

Islandicin (**12**): Red solid; IR (ATR) *ν_max_:* 3376, 3139, 1659, 1523, 1444, 1336, 1273 cm^−1^; ^1^H NMR (400 MHz, CDCl_3_) δ 13.49 (OH-1), 12.33 (OH-8), 12.29 (OH-4), 7.89 (1H, dd, *J =* 7.6, 1.1 Hz, H-5), 7.69 (1H, t, *J =* 8.4 Hz, H-6), 7.31 (1H, dd, *J =* 8.4, 1.1 Hz, H-7), 7.16 (1H, q, *J =* 0.9 Hz, H-2), 2.38 (3H, d, *J =* 1.0 Hz, CH_3_-3); ^13^C NMR (100 MHz, CDCl_3_) δ 190.6 (C-9), 186.7 (C-10), 162.7 (C-8), 158.0 (C-4), 157.9 (C-1), 142.0 (C-3), 136.8 (C-6), 133.8 (C-10a), 129.2 (C-2), 124.7 (C-7), 119.5 (C-5), 116.4 (C-8a), 111.8 (C-4a), 110.9 (C-9a), 16.6 (CH_3_-3).

### 3.4. HDAC Inhibitory Activity Assay

The HDAC inhibitory activity was determined by Fluor-de-Lys HDAC activity assay kit (Biomol, Enzo Life Sciences International, Inc., USA). The assay was carried out according to the manufacturer’s instructions. In brief, preparation of the recombinant HeLa nuclear extract was performed by diluted in assay buffer and added to a microliter plate. The Fluor de Lys^TM^ substrate was diluted with assay buffer. HDAC reaction was started by adding the substrate to each well and incubated at 37 °C for 10 min. The reaction was stopped by the addition of a developer and then incubated at room temperature for 10 min. After 10 min of incubation, the samples were monitored by SpectraMax M5 (Molecular Devices, USA). The fluorescence was measured at excitation wavelength 360 nm and emitted light 460 nm. Trichostatin A (TSA) was used as the positive control. All experiments were carried out in triplicate.

### 3.5. In Silico Physicochemical Properties

SwissADME web server [23] was used to assess the physicochemical properties for determination of the good drug candidates. In this study, the physicochemical parameters (molecular weight, topological polar surface area (TPSA), number of rotatable bonds, number of hydrogen-bond acceptors, and number of hydrogen-bond donors), and lipophilicity were checked for the evaluated compounds. Together, the Lipinski’s and Veber’s rules were used to verify the drug-likeness profile.

### 3.6. Molecular Docking

Molecular docking was performed using AutoDock 4.2 to calculate the binding free energies and to obtain the best orientation of selected compounds with HDAC1, HDAC2, HDAC4, HDAC7, and HDAC8 (PDB entry code: 4BKX [24], 3MAX [25], 2VQW [26], 3C0Z [27], and 1T64 [28], respectively) template. For all of the docking calculations, Lamarkian genetic algorithm search (LGA) was used. Polar hydrogens and Gasteiger charges were assigned by using AutoDockTools (ADT) [29]. All water and non-interacting ions, as well as, ligands were removed. Atomic salvation parameters, based on the Stouten model and fragmental volumes, were added in accordance with the AutoDock force field [30,31]. The grid box site of 60 × 60 × 60 points with grid spacing of 0.375 Å. The cartesian coordination grid box measures 11.058 × 7.784 × 31.524, the center base of the crystal ligand. The AutoGrid 4.2 program was used to generate the grid map files. Maximum energy evaluations of 2.5 × 106 steps were performed with a population size of 200 ligand orientations while the total independent runs were fixed to 200. The final docked structure, RMSD from the bound crystal structure, docked energy, and predicted free energy of binding were used to analyze its interaction with the active site. The best orientations with the lowest docked energies were visualized for their interactions by using LigPlot+ software [32].

### 3.7. MTT Assay

The MTT reduction assay was performed with non-cancer (Vero), human cervical cancer (HeLa), human lung carcinoma (A549) and human breast adenocarcinoma cancer (MCF-7) cell lines according to the method previously described [33,34,35]. Briefly, cells were seeded in a 96-well plate and incubated for 24 h. The cells were treated with the selected compounds and incubated at 37 °C in CO_2_ for 24 h, 48 h, and 72 h. After incubation, the culture medium was exchanged with 110 μL of MTT (0.5 mg/mL in PBS medium) and incubated for 2 h. The amount of MTT formazan product was determined after dissolved in DMSO by measuring its absorbance with a microplate reader (Bio-Rad Laboratories, USA) at a test wavelength of 550 nm and a reference wavelength of 655 nm. The cell viability was expressed as a percentage to the viable cells of control culture condition, and IC_50_ values of each group were calculated.

## 4. Conclusions

Four new anthraquinones, ventilanones L–O (**1**–**4**), together with eight known anthraquinones (**5**–**12**) were isolated from the roots of *Ventilago denticulata*. Among the known compounds, this is the first report of **5** as a naturally occurring anthraquinone. Additionally, **8** was first isolated from the genus *Ventilago*, whereas **11** was first isolated from the plant *V. denticulata*. Physicochemical properties analysis revealed that **1**–**4** displayed no violation of Lipinski’s rules and suggested that they have good drug-likeness properties. The molecular docking study demonstrated that **2** is well embedded into HDAC4 enzyme active site, generating hydrogen-bonds to amino acids and ionic interaction to ZBG. The antiproliferative activity evaluation showed that **2** exhibited moderate toxicity against HeLa and A549 cell lines but showed nontoxic to normal cells.

## Figures and Tables

**Table 1:** The ^1^H NMR data (400 MHz) of compounds **1**–**4**.

Position	*δ*_H_ Multiplicity (*J* in Hz)			
	1 (DMSO-*d*_6_)	2 (CDCl_3_)	3 (DMSO-*d*_6_)	4 (CDCl_3_)
2		7.01 dd (1.7, 0.7)		7.66 s
4	7.52 s	7.54 dd (1.7, 0.7)	7.47 d (0.6)	
5			7.22 d (2.4)	7.81 dd (7.5, 1.1)
6				7.67 dd (8.2, 7.5)
7	6.68 s		6.81 d (2.4)	7.29 dd (8.2, 1.1)
1-COOCH_3_				3.98 s
2-COOCH_3_	3.89 s			
3-CH_3_	2.36 s	2.42 dd (0.7, 0.7)	2.25 d (0.6)	2.44 s
5-CH_3_	2.45 s	2.59 s		
6-OCH_3_		3.97 s		
7-OCH_3_		4.01 s		
8-OCH_3_			3.92 s	
1-OH	12.28 s	11.91 s	13.65, s	
4-OH				12.34 s
8-OH	12.59 s	13.03 s		11.94 s

**Table 2:** The ^13^C NMR data (100 MHz) of compounds **1**–**4**.

Position	δ_C_, Type			
	1 (DMSO-*d*_6_)	2 (CDCl_3_)	3 (DMSO-*d*_6_)	4 (CDCl_3_)
1	157.3, C	162.2, C	149.6, C	133.7, C
2	127.6, C	123.4, CH	150.6, C	121.7, CH
3	144.2, C	149.1, C	130.1, C	129.2, C
4	120.4, CH	121.2, CH	121.4, CH	159.9, C
5	124.7, C	126.8, C	106.9, CH	120.4, CH
6	165.2, C	158.5, C	163.4, C	137.6, CH
7	106.8, CH	145.7, C	104.3, CH	125.1, CH
8	163.2, C	157.0, C	164.6, C	162.8, C
9	189.4, C	192.2, C	186.9, C	192.5, C
10	183.4, C	183.8, C	180.8, C	181.6, C
4a	134.5, C	135.0, C	122.4, C	146.6, C
8a	109.1, C	113.6, C	112.7, C	115.9, C
9a	113.7, C	113.7, C	115.0, C	114.3, C
10a	131.5, C	131.0, C	137.4, C	133.6, C
1-COO				166.7, C
2-COO	166.2, C			
3-CH_3_	19.7, CH_3_	22.5, CH_3_	16.3, CH_3_	20.7, CH_3_
5-CH_3_	13.0, CH_3_	14.2, CH_3_		
1-COOCH_3_				53.3, CH_3_
2-COOCH_3_	52.6, CH_3_			
6-OCH_3_		61.2, CH_3_		
7-OCH_3_		61.4, CH_3_		
8-OCH_3_			56.3, CH_3_	

**Table 3:** HDAC inhibitory activity of compounds **2**, **6**, and **9** at 40 μg/mL.

Compounds	% Inhibition
Ventilanone M (**2**)	61.27
Physcion (**6**)	30.15
Emodin (**9**)	17.30
TSA	86.10 *

* (at 2.5 μM).

**Table 4:** Physicochemical properties of **1**–**4** by in silico analysis (SwissADME program).

Compounds	Physicochemical Properties					
	MW (g/mol)	TPSA ^a^ (Å^2^)	Num. Rotatable Bonds	Num. H-Bond Acceptors	Num. H-Bond Donors	Log Po/w ^b^
**1**	342.30	121.13	2	7	3	3.05
**2**	328.32	93.06	2	6	2	3.65
**3**	300.26	104.06	1	6	3	2.60
**4**	312.27	100.90	2	6	2	3.00

^a^ TPSA topological polar surface area, ^b^ Log Po/w octanol/water partition coefficient.

**Table 5:** In silico histone deacetylase inhibitory activity of **2**.

Compounds	Class I HDACs						Class II HDACs			
	HDAC1		HDAC2		HDAC8		HDAC4		HDAC7	
	ΔG *	Ki **	ΔG *	Ki **	ΔG *	Ki **	ΔG *	Ki **	ΔG *	Ki **
2	−5.91	46.81	−6.12	32.92	−6.46	18.42	−6.85	9.49	−6.46	5.29
SAHA	−6.23	27.28	−7.43	3.55	−7.52	3.09	−5.20	152.69	−4.79	228.88

* (kcal/mol), ** (μM).

**Table 6:** Antiproliferative activity of the HDAC inhibitors against cancer cell lines.

Cell Lines	IC_50_ Values (Mean ± SD; *n* = 3; μM)					
	Compound 2			Cisplatin		
	24 h	48 h	72 h	24 h	48 h	72 h
HeLa cells	240.46 ± 8.14	190.08 ± 2.97	160.87 ± 2.08	17.07 ± 1.00	9.97 ± 0.34	6.45 ± 0.13
A549 cells	>300	203.17 ± 6.56	177.32 ± 5.32	65.36 ± 8.11	11.44 ± 1.99	5.06 ± 0.01
MCF−7 cells	>300	>300	>300	29.17 ± 4.48	13.75 ± 1.81	10.42 ± 0.85
Vero cells	>300	>300	273.47 ± 3.49	42.85 ± 2.38	12.36 ± 0.63	6.55 ± 0.81

## Data Availability

Not applicable.

## Supplementary Materials

The following are available online: Figures S1–S7: NMR and MS spectra and spectral data of compound **1**, Figures S8–S14: NMR and MS spectra and spectral data of compound **2**, Figures S15–S21: NMR and MS spectra and spectral data of compound **3**, Figures S22–S28: NMR and MS spectra and spectral data of compound **4**, Figures S29–S48: NMR spectra and spectral data of compounds **5**–**12**.

## Sample Availability

Samples of the compounds **1**–**12** are available from the authors.
