# Supplementary material for: Four New Anthraquinones with Histone Deacetylase Inhibitory Activity from Ventilago denticulata Roots
Source: Molecules. 2022 Feb 6;27(3):1088. doi: 10.3390/molecules27031088 (PMC8839831; doi:10.3390/molecules27031088)
Supplement: Supplementary file 1 [file molecules-27-01088-s001.zip › molecules-1560424-supplementary.pdf]

SUPPLEMENTARY MATERIAL

Four New Anthraquinones with Histone Deacetylase Inhibitory Activity from *Ventilago denticulata* Roots

Nattika Hangsamai [1], Kanokwan Photai [1], Thidathep Mahaamnart [1], Somdej Kanokmedhakul [1], Kwanjai Kanokmedhakul [1] , Thanaset Senawong [2], Siripit Pitchuanchom [3] and Mongkol Nontakitticharoen [1,*]

[1] Natural Products Research Unit, Department of Chemistry and Center of Excellence for Innovation in Chemistry, Faculty of Science, Khon Kaen University, Khon Kaen 40002, Thailand; nattika_h@kkumail.com (N.H.); kanokwan_kp@kkumail.com (K.P.); m_thidathep@kkumail.com (T.M.); somdej@kku.ac.th (S.K.); kwanjai@kku.ac.th (K.K.)

[2] Natural Products Unit, Department of Biochemistry, Faculty of Science, Khon Kaen University, Khon Kaen 40002, Thailand; sthanaset@kku.ac.th

[3] Department of Chemistry and Center of Excellence for Innovation in Chemistry, Faculty of Science, Mahasarakham University, Maha Sarakham 44150, Thailand; siripit.p@msu.ac.th

* Correspondence: mongbu@kku.ac.th; Tel.: +66 43–009–700 ext 42174

Contents:

	Page
**Figure S1.** ^{1}H NMR (400 MHz, $CDCl_3$) spectrum of compound **1**	4
**Figure S2.** ^{13}C NMR (100 MHz, $CDCl_3$) spectrum of compound **1**	4
**Figure S3.** COSY ($CDCl_3$) spectrum of compound **1**	5
**Figure S4.** HSQC ($CDCl_3$) spectrum of compound **1**	5
**Figure S5.** HMBC ($CDCl_3$) spectrum of compound **1**	6
**Figure S6.** NOESY ($CDCl_3$) spectrum of compound **1**	6
**Figure S7.** MS spectrum of compound **1**	7
**Figure S8.** ^{1}H NMR (400 MHz, $CDCl_3$) spectrum of compound **2**	7
**Figure S9.** ^{13}C NMR (400 MHz, $CDCl_3$) spectrum of compound **2**	8
**Figure S10.** COSY ($CDCl_3$) spectrum of compound **2**	8
**Figure S11.** HSQC ($CDCl_3$) spectrum of compound **2**	9
**Figure S12.** HMBC ($CDCl_3$) spectrum of compound **2**	9
**Figure S13.** NOESY ($CDCl_3$) spectrum of compound **2**	10
**Figure S14.** MS spectrum of compound **2**	10
**Figure S15.** ^{1}H NMR (400 MHz, $CDCl_3$) spectrum of compound **3**	11
**Figure S16.** ^{13}C NMR (100 MHz, $CDCl_3$) spectrum of compound **3**	11
**Figure S17.** COSY ($CDCl_3$) spectrum of compound **3**	12
**Figure S18.** HSQC ($CDCl_3$) spectrum of compound **3**	12
**Figure S19.** HMBC ($CDCl_3$) spectrum of compound **3**	13
**Figure S20.** NOESY ($CDCl_3$) spectrum of compound **3**	13
**Figure S21.** MS spectrum of compound **3**	14
**Figure S22.** ^{1}H NMR (400 MHz, $CDCl_3$) spectrum of compound **4**	14
**Figure S23.** ^{13}C NMR (100 MHz, $CDCl_3$) spectrum of compound **4**	15
**Figure S23.** COSY ($CDCl_3$) spectrum of compound **4**	15
**Figure S25.** HSQC ($CDCl_3$) spectrum of compound **4**	16
**Figure S26.** HMBC ($CDCl_3$) spectrum of compound **4**	16
**Figure S27.** NOESY ($CDCl_3$) spectrum of compound **4**	17
**Figure S28.** MS spectrum of compound **4**	17
**Figure S29.** ^{1}H NMR (400 MHz, $CDCl_3$) spectrum of 7-Methoxyphyscion **5**	18

Figure S30. ^{13}C NMR (100 MHz, $CDCl_3$) spectrum of 7-Methoxyphyscion **5** 18
Figure S31. MS spectrum of 7-Methoxyphyscion **5** 19
Figure S32 ^{1}H NMR (400 MHz, $CDCl_3$) spectrum of Physcion **6** 19
Figure S33 ^{13}C NMR (100 MHz, $CDCl_3$) spectrum of Physcion **6** 20
Figure S34. MS spectrum of Physcion **6** 20
Figure S35. ^{1}H NMR (400 MHz, $CDCl_3$) spectrum of Chrysophanol **7** 21
Figure S36. ^{13}C NMR (100 MHz, $CDCl_3$) spectrum of Chrysophanol **7** 21
Figure S37. MS spectrum of Chrysophanol **7** 22
Figure S38. ^{1}H NMR (400 MHz, $CDCl_3$) spectrum of 2-Methoxychrysophanol **8** 22
Figure S39. ^{13}C NMR (100 MHz, $CDCl_3$) spectrum of 2-Methoxychrysophanol **8** 23
Figure S40. ^{1}H NMR (400 MHz, $CDCl_3$+CD_3OD) spectrum of Emodin **9** 23
Figure S41. ^{13}C NMR (100 MHz, $CDCl_3$+CD_3OD) spectrum of Emodin **9** 24
Figure S42. MS spectrum of Emodin **9** 24
Figure S43. ^{1}H NMR (400 MHz, $CDCl_3$) spectrum of Emodin-6,8-dimethyl ether **10** 25
Figure S44. ^{13}C NMR (100 MHz, $CDCl_3$) spectrum of Emodin-6,8-dimethyl ether **10** 25
Figure S45. ^{1}H NMR (400 MHz, $CDCl_3$) spectrum of 2-Hydroxyemodin-1-methyl ether **11** 26
Figure S46. ^{13}C NMR (400 MHz, $CDCl_3$) spectrum of 2-Hydroxyemodin-1-methyl ether **11** 26
Figure S47. ^{1}H NMR (400 MHz, $CDCl_3$) spectrum of Islandicin **12** 27
Figure S48. ^{13}C NMR (100 MHz, $CDCl_3$) spectrum of Islandicin **12** 27

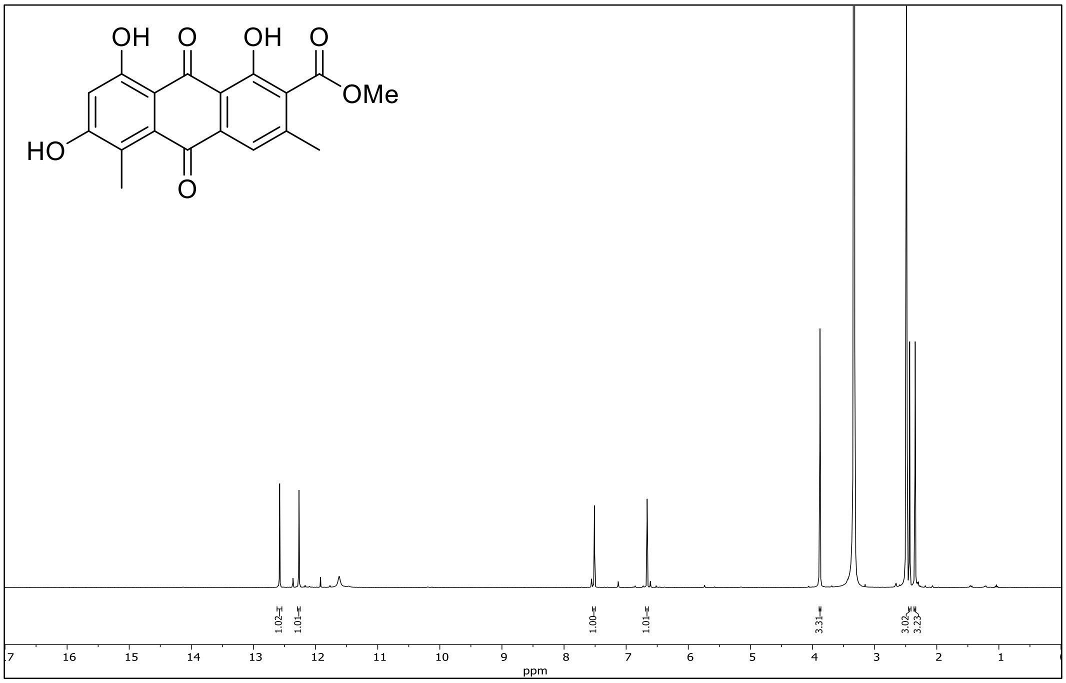

Figure S1. ^{1}H NMR (400 MHz, $CDCl_3$) spectrum of compound **1**

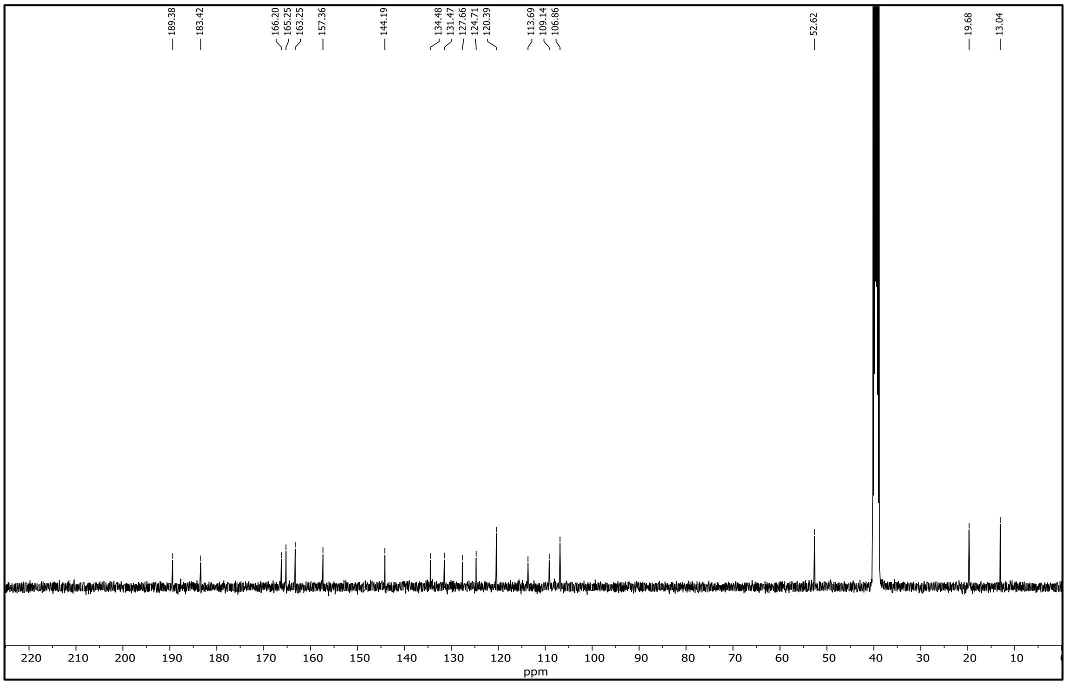

Figure S2. ^{13}C NMR (100 MHz, $CDCl_3$) spectrum of compound **1**

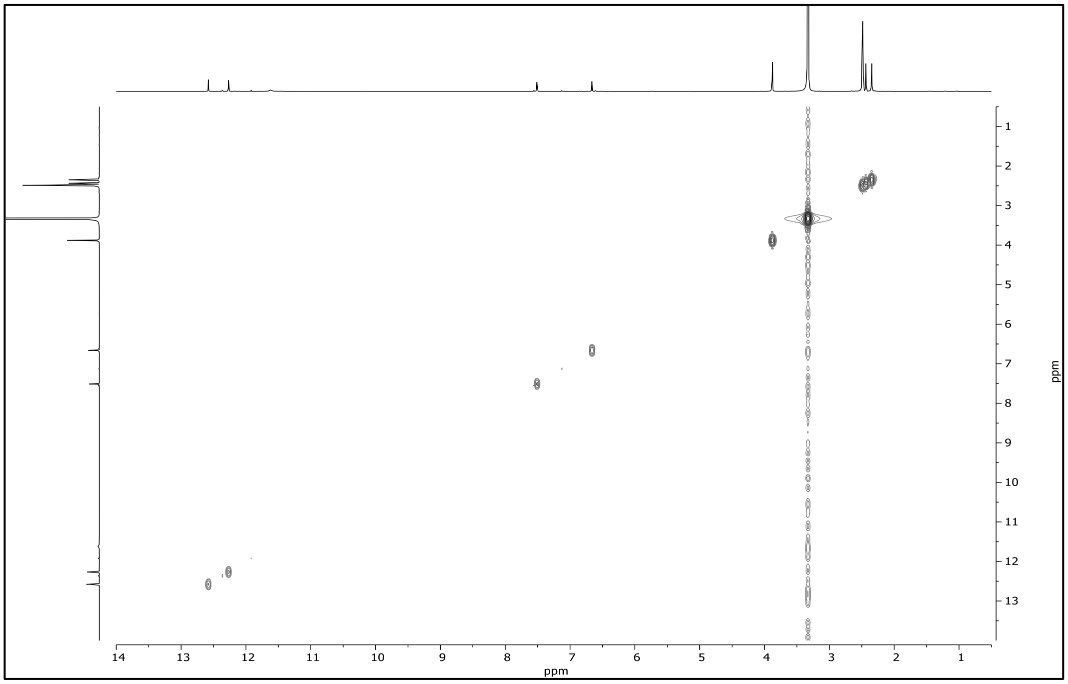

Figure S3. COSY ($CDCl_3$) spectrum of compound **1**

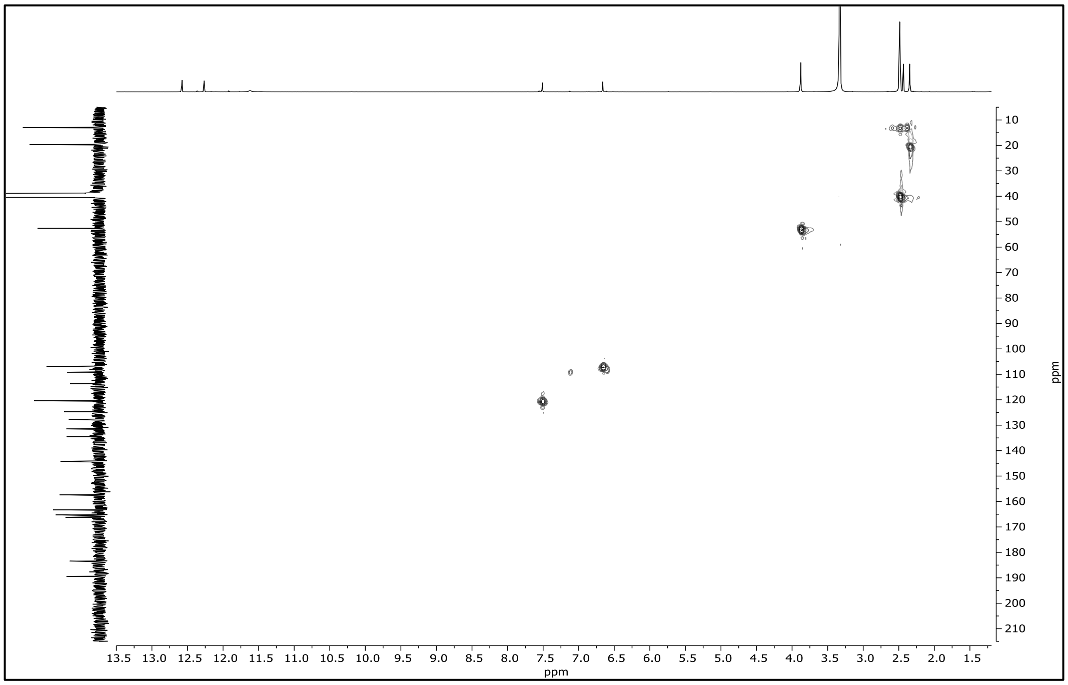

Figure S4. HSQC ($CDCl_3$) spectrum of compound **1**

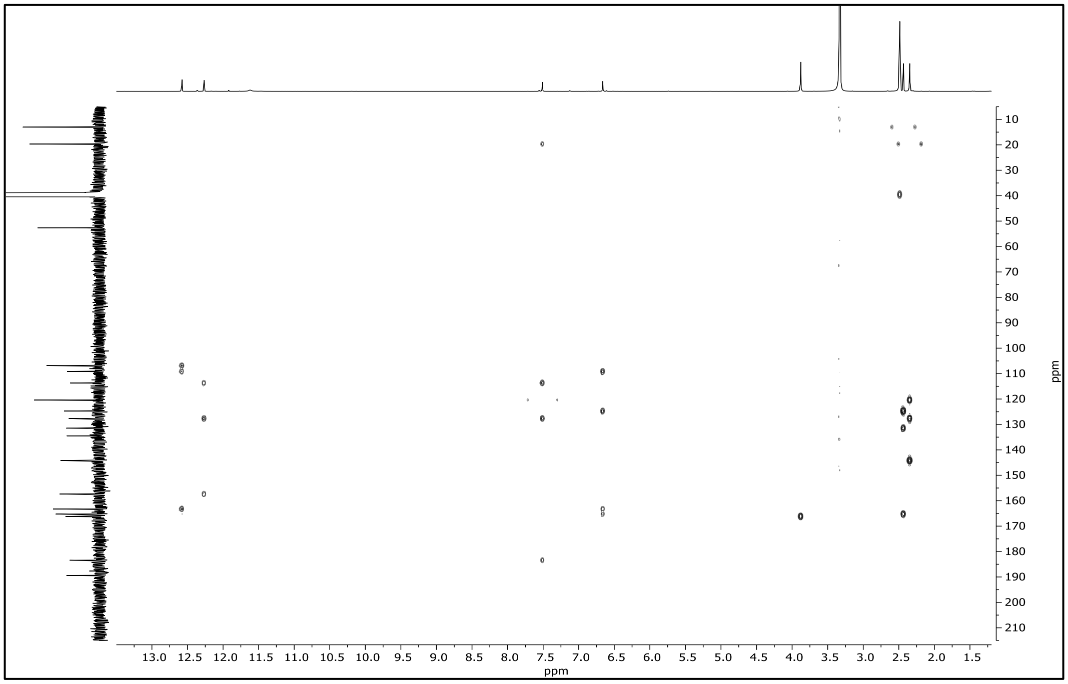

Figure S5. HMBC ($CDCl_3$) spectrum of compound **1**

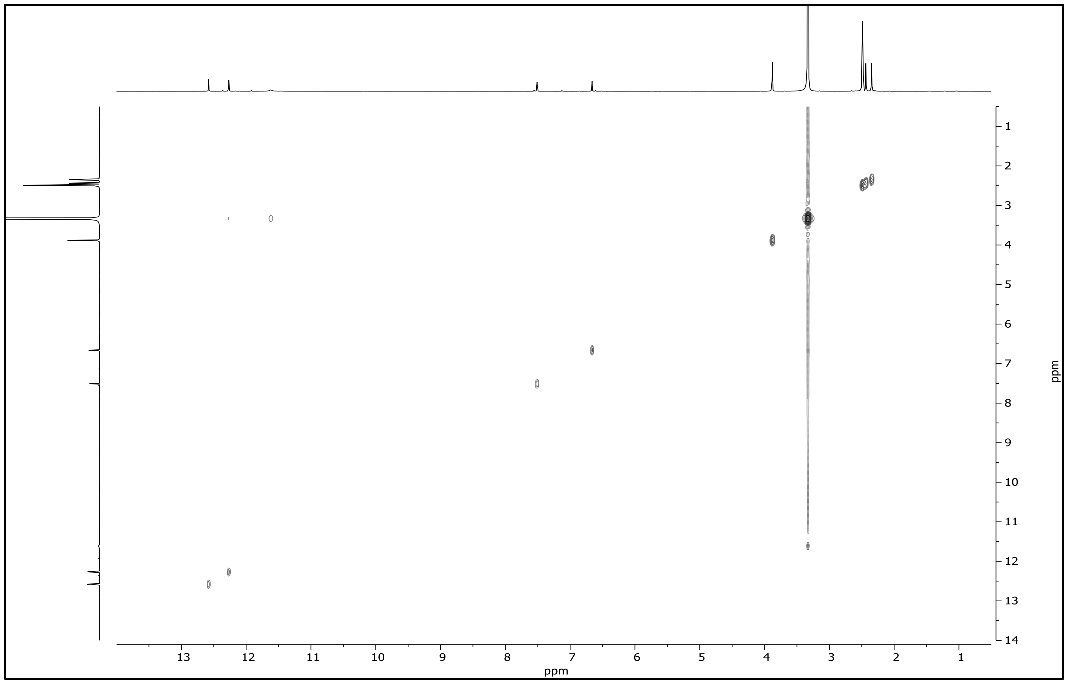

Figure S6. NOESY ($CDCl_3$) spectrum of compound **1**

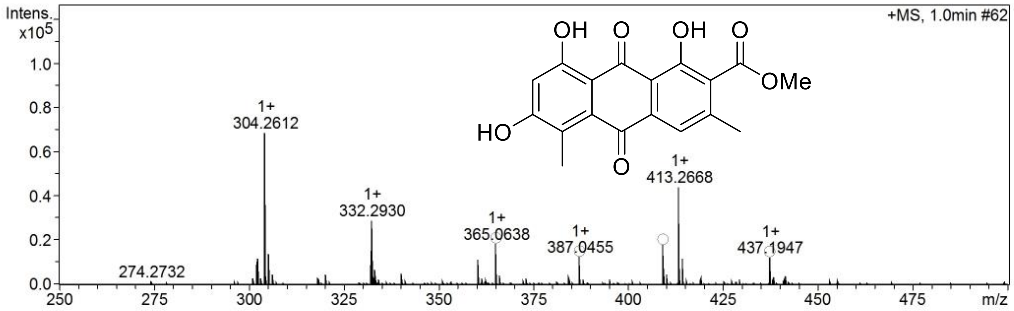

Meas. m/z	#	Ion Formula	m/z	|err| [mDa]	err [ppm]	Mean err [ppm]	rdb	N-Rule	e¯ Conf	mSigma
365.063805	1	C20H13O7	365.065579	1.8	4.9	4.9	14.5	ok	even	3.4
	2	C23H9O5	365.044450	19.4	53.0	-52.9	19.5	ok	even	14.0
	3	C24H13O4	365.080835	17.0	-46.6	46.7	18.5	ok	even	19.3
	4	C16H13O10	365.050323	13.5	-36.9	-36.9	10.5	ok	even	23.3
	5	C27H9O2	365.059706	4.1	11.2	-11.1	23.5	ok	even	35.0
	1	C22H14NaO4	365.078430	14.6	40.1	40.1	15.5	ok	even	8.4
	2	C18H14NaO7	365.063173	0.6	1.7	-1.7	11.5	ok	even	13.1
	3	C25H10NaO2	365.057300	6.5	-17.8	-17.7	20.5	ok	even	24.0
	4	C14H14NaO10	365.047917	15.9	43.5	-43.5	7.5	ok	even	34.1

Figure S7. MS spectrum of compound **1**

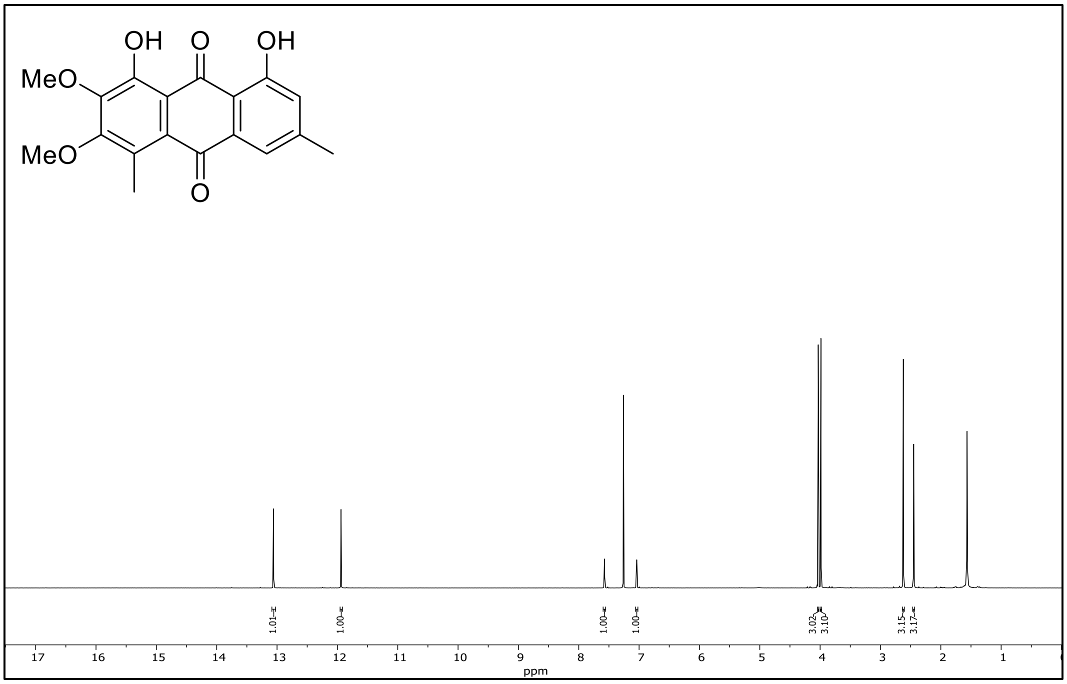

Figure S8. ^{1}H NMR (400 MHz, $CDCl_3$) spectrum of compound **2**

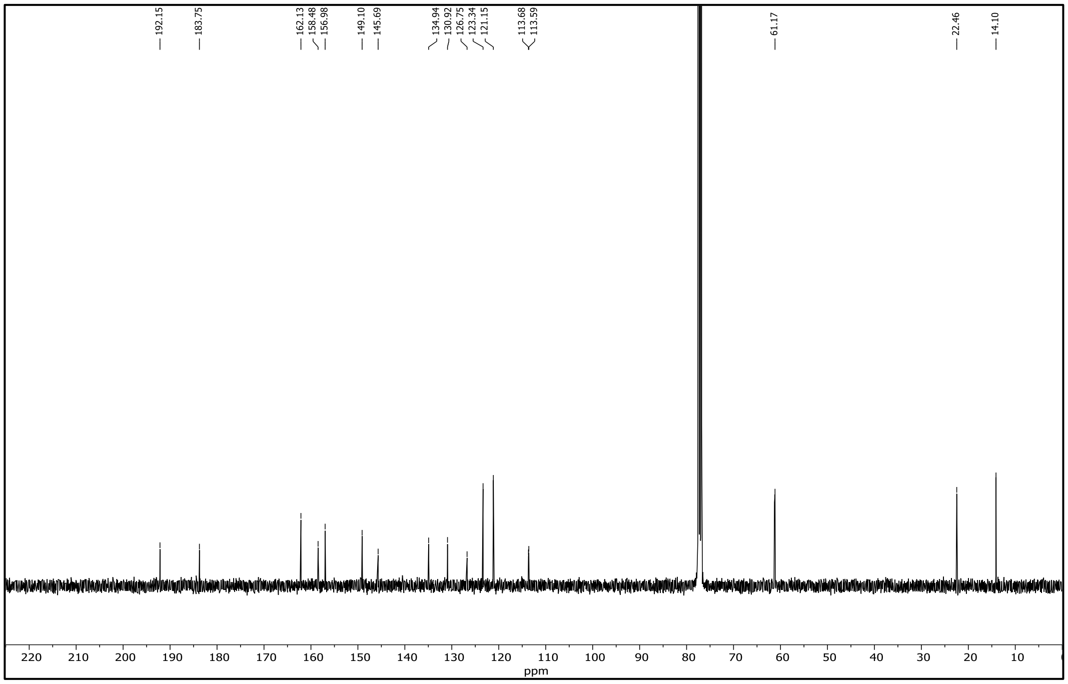

Figure S9. ^{13}C NMR (400 MHz, $CDCl_3$) spectrum of compound **2**

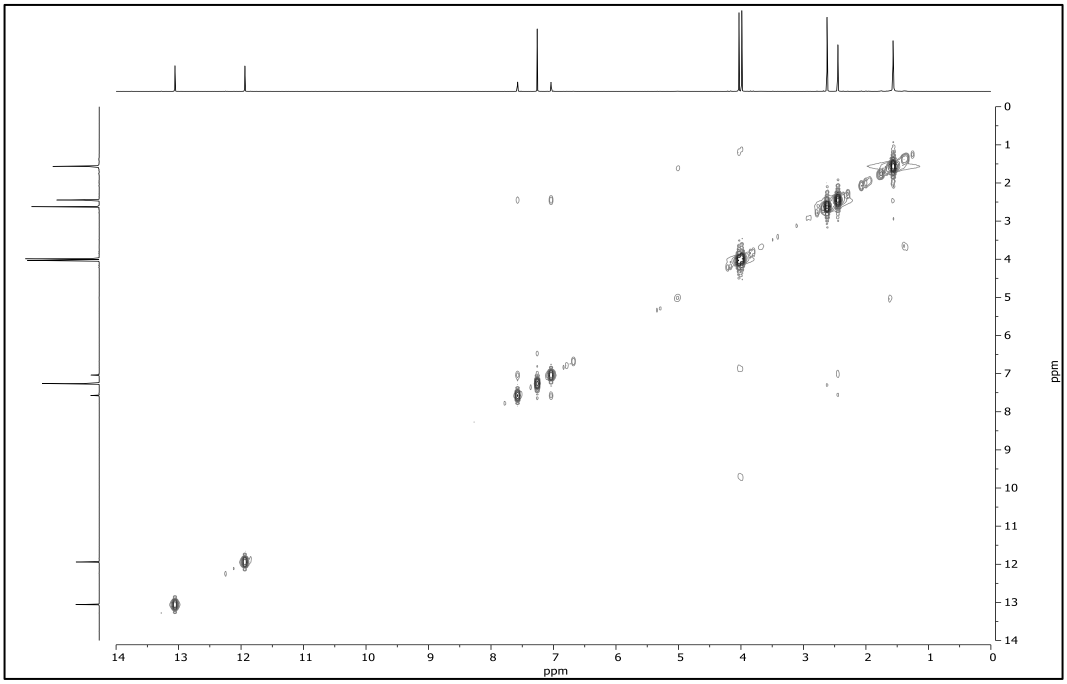

Figure S10. COSY ($CDCl_3$) spectrum of compound **2**

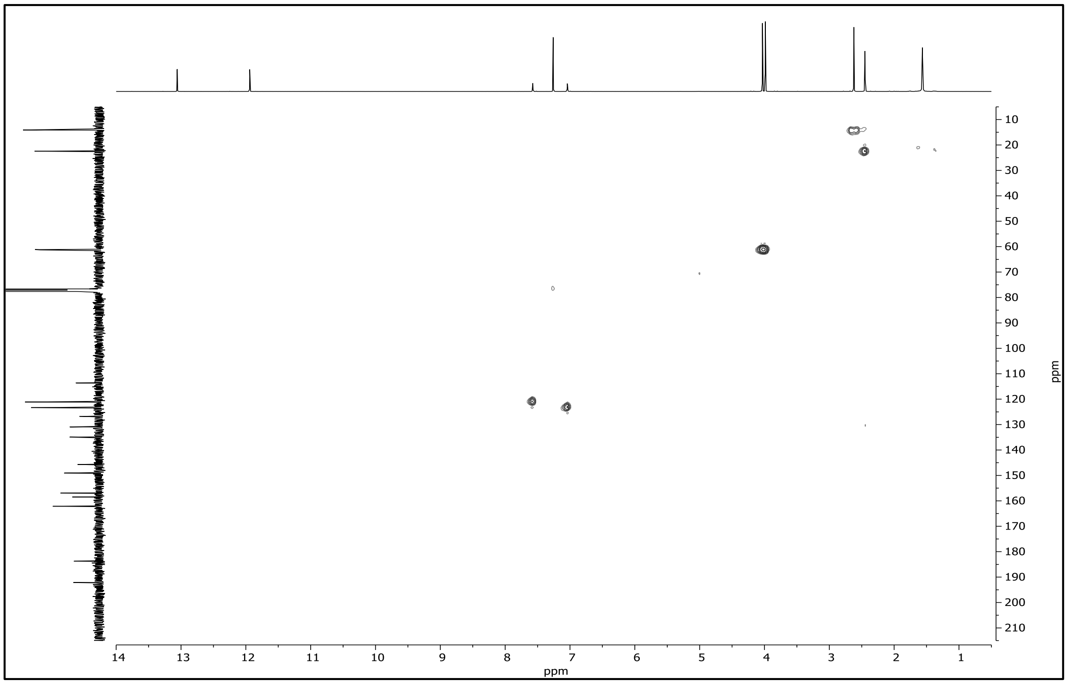

Figure S11. HSQC ($CDCl_3$) spectrum of compound **2**

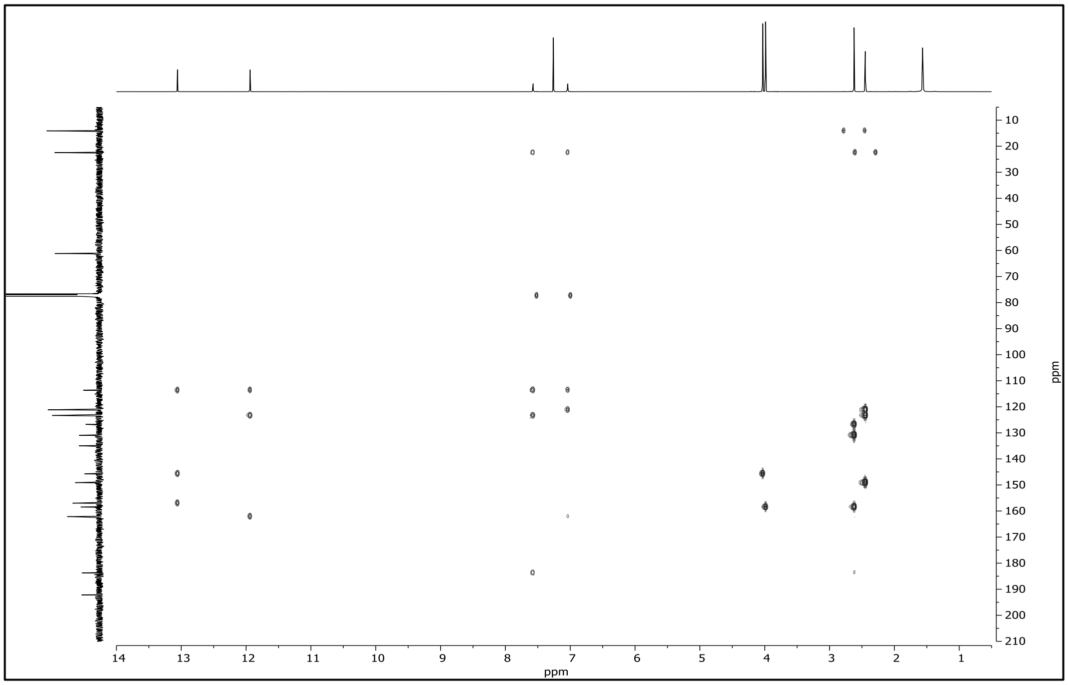

Figure S12. HMBC ($CDCl_3$) spectrum of compound **2**

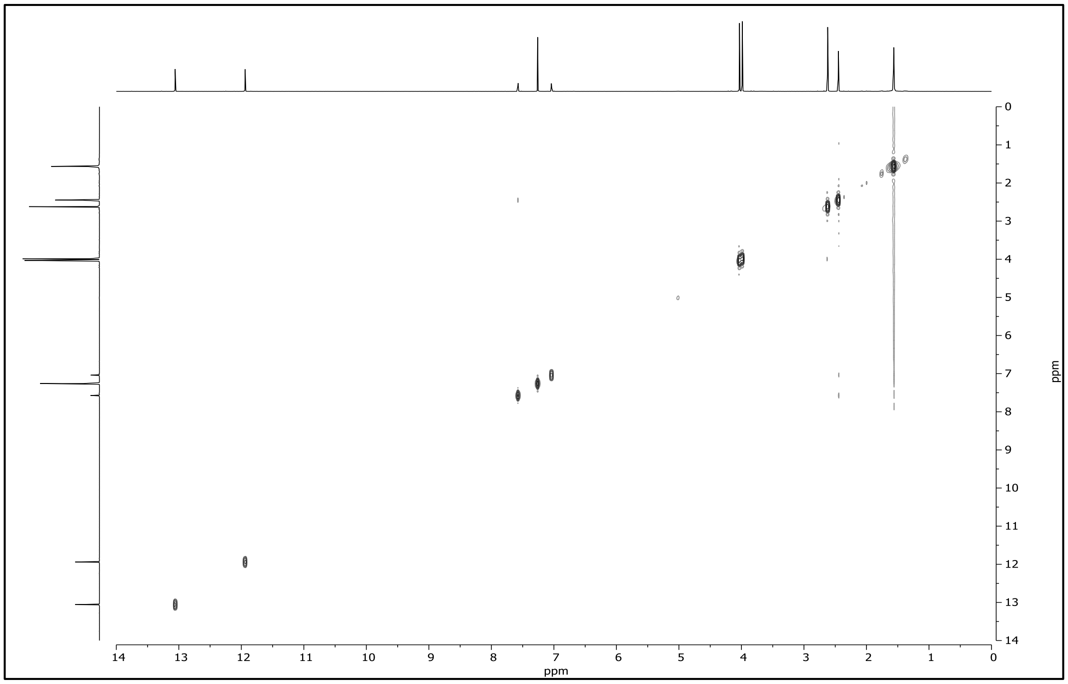

Figure S13. NOESY ($CDCl_3$) spectrum of compound **2**

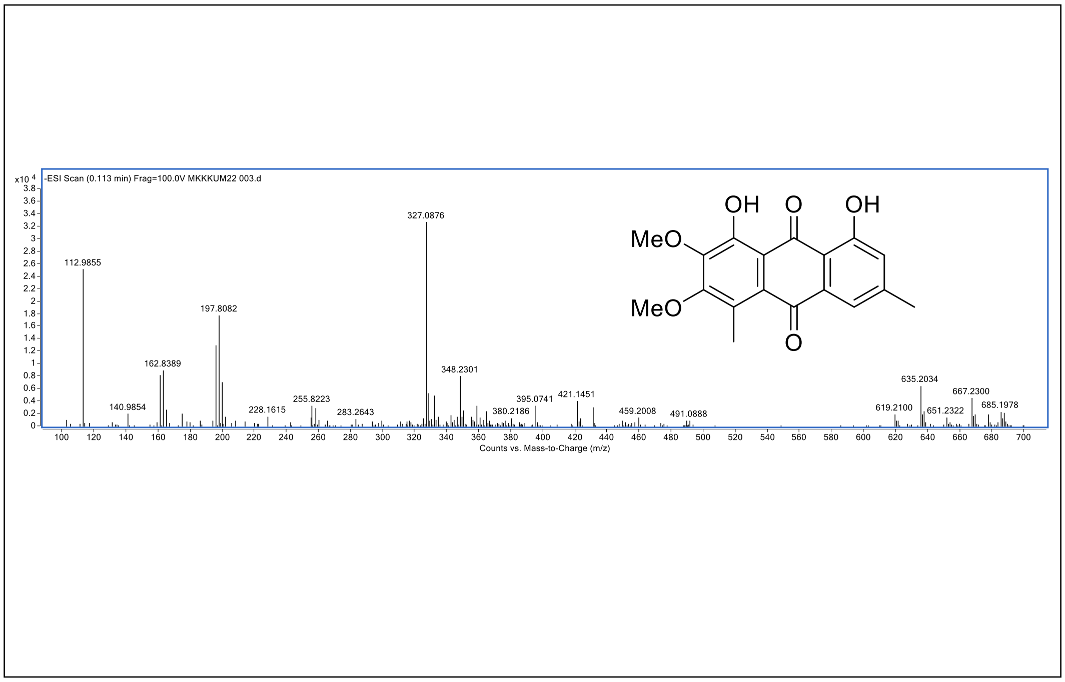

Figure S14. MS spectrum of compound **2**

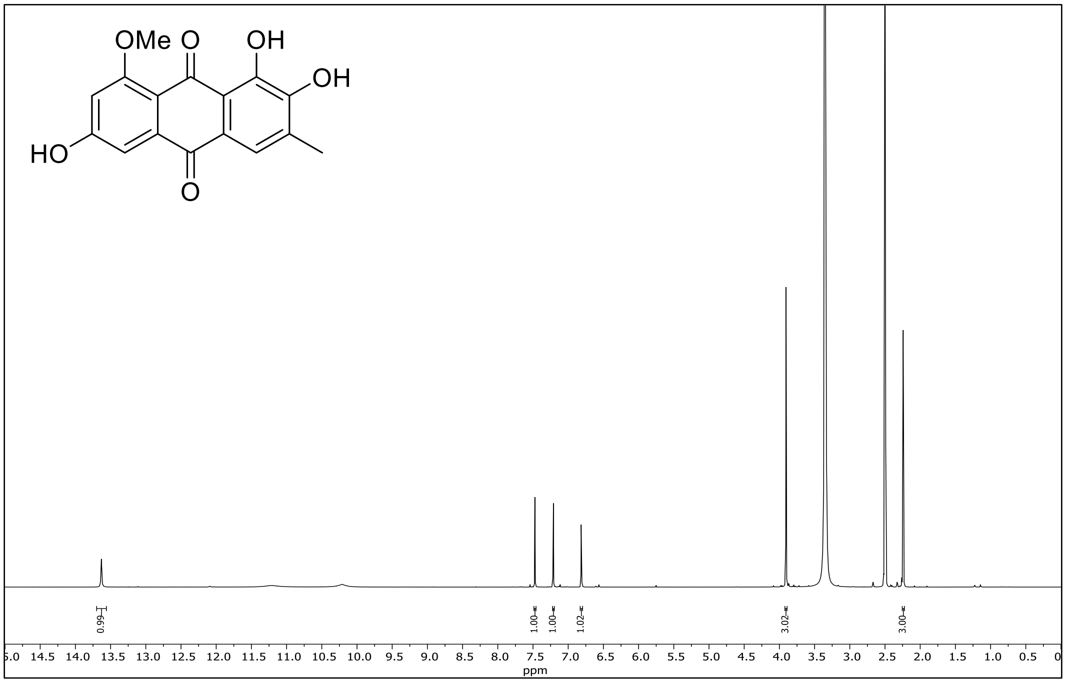

Figure S15. ^{1}H NMR (400 MHz, $CDCl_3$) spectrum of compound **3**

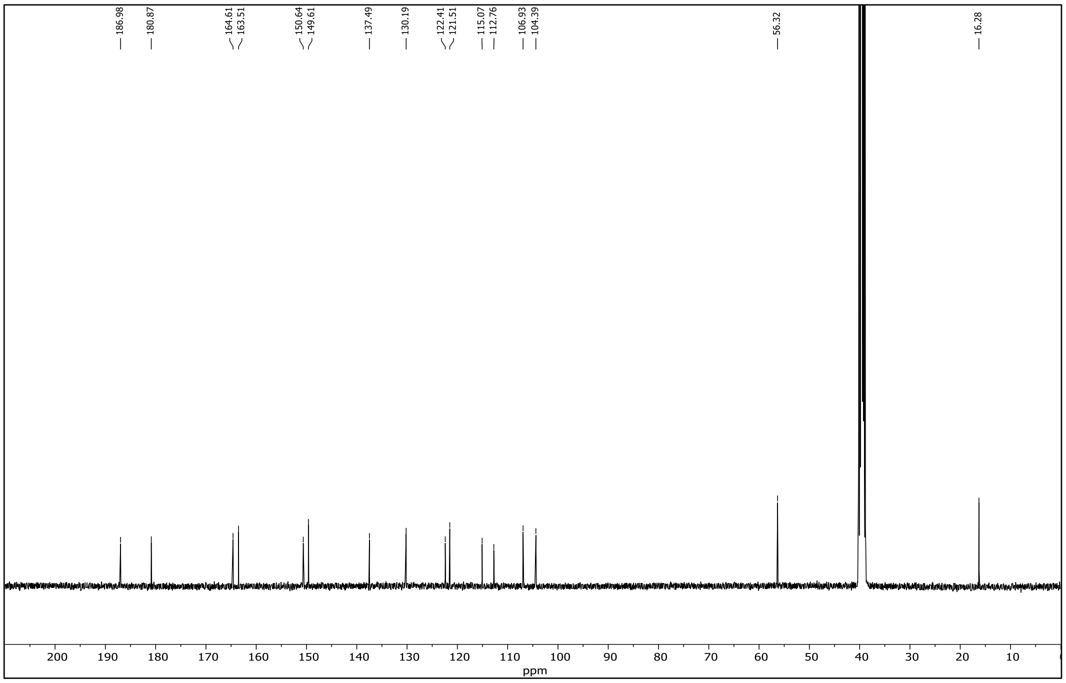

Figure S16. ^{13}C NMR (100 MHz, $CDCl_3$) spectrum of compound **3**

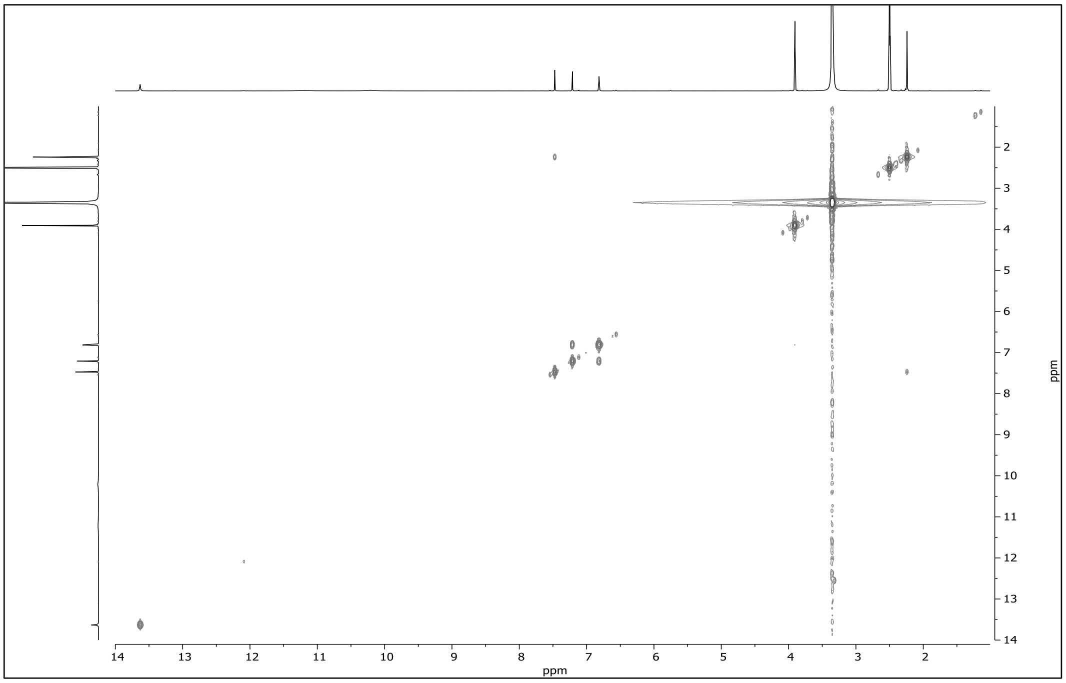

Figure S17. COSY ($CDCl_3$) spectrum of compound **3**

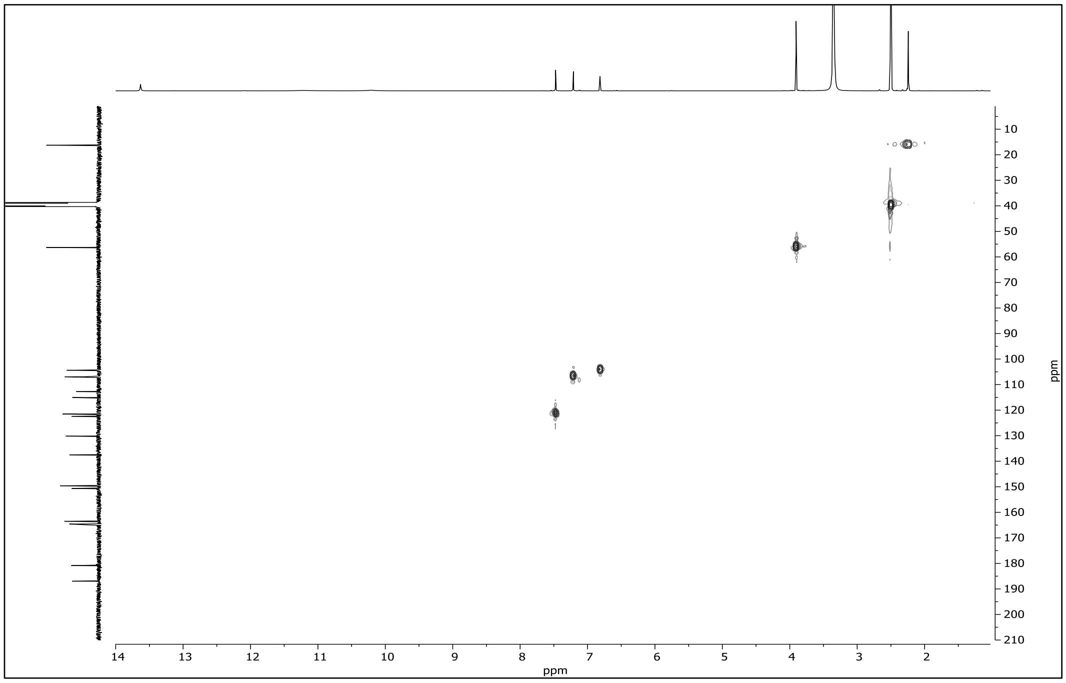

Figure S18. HSQC ($CDCl_3$) spectrum of compound **3**

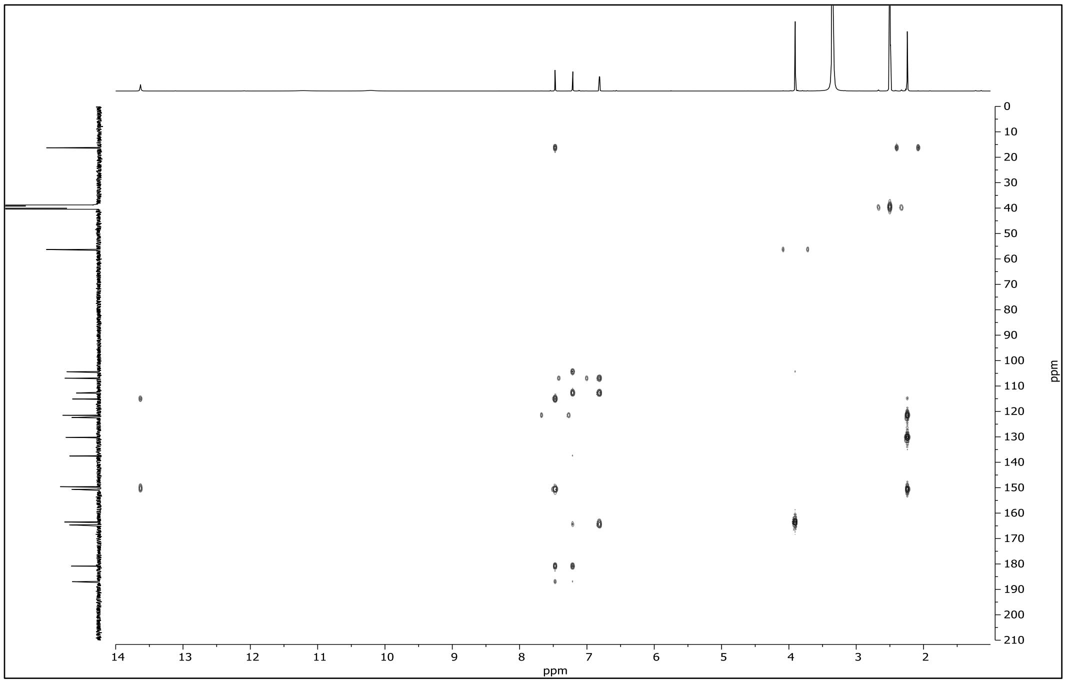

Figure S19. HMBC ($CDCl_3$) spectrum of compound **3**

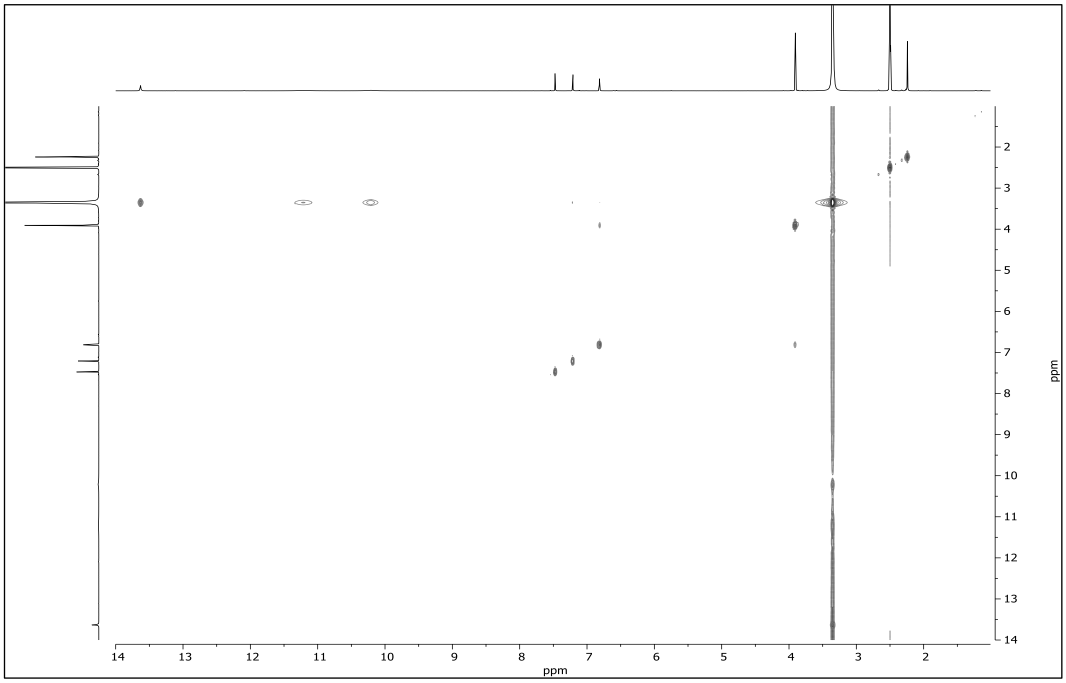

Figure S20. NOESY ($CDCl_3$) spectrum of compound **3**

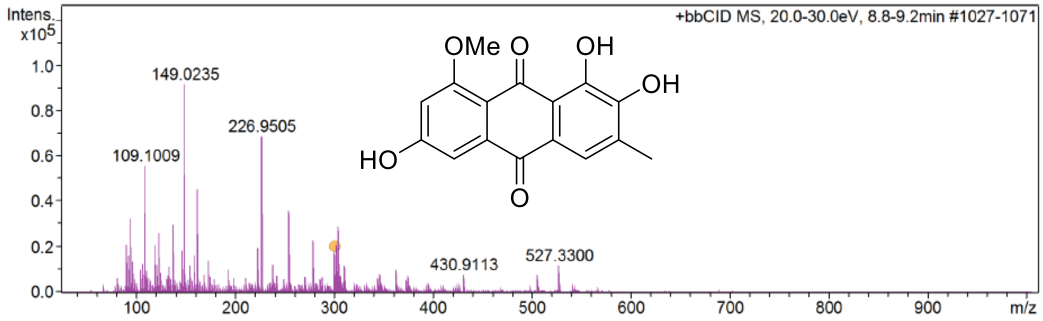

Meas. m/z	#	Ion Formula	m/z	err [ppm]	mSigma	# mSigma	Score	rdb	e⁻ Conf	N-Rule
301.0690	1	C14H11N3O5	301.0693	1.0	9.6	1	100.00	11.5	odd	ok
	2	C16H13O6	301.0707	5.5	11.7	2	44.93	11.0	even	ok
	3	C12H9N6O4	301.0680	-3.5	11.9	3	65.43	12.0	even	ok
	4	C13H5N10	301.0693	1.0	17.0	4	86.67	17.0	even	ok

Figure S21. MS spectrum of compound **3**

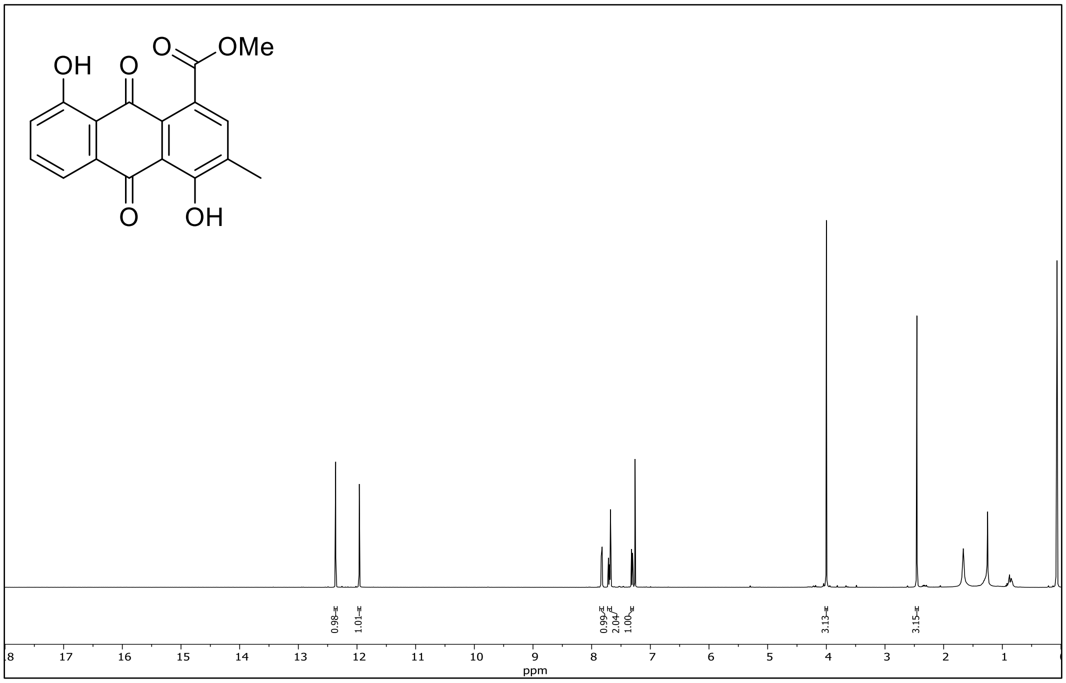

Figure S22. ^{1}H NMR (400 MHz, $CDCl_3$) spectrum of compound **4**

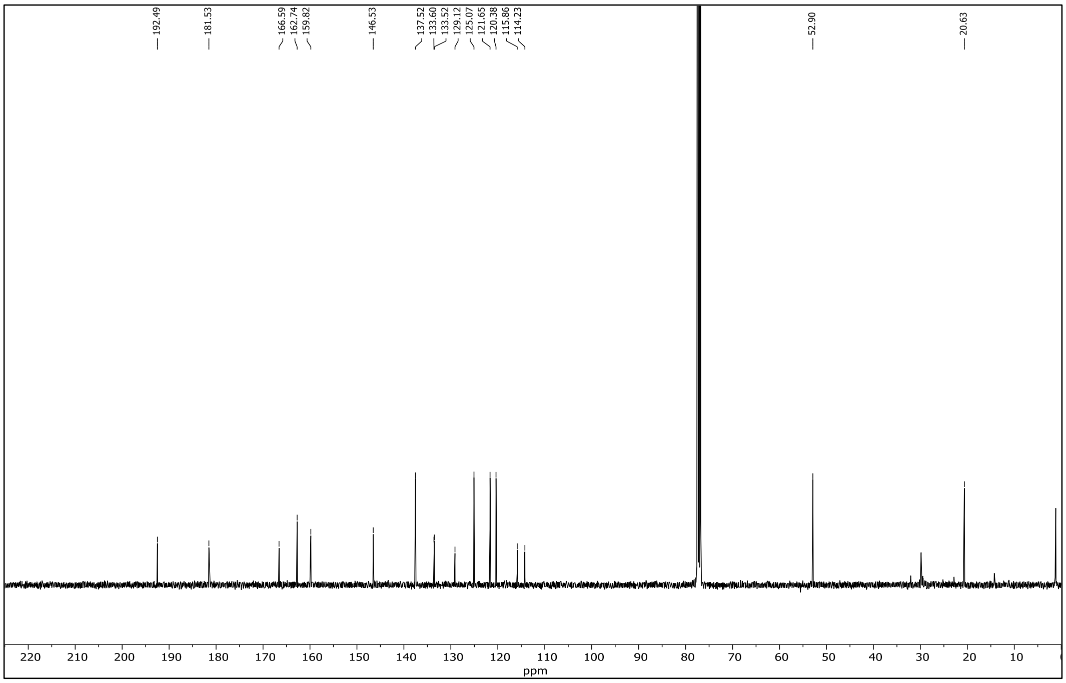

Figure S23. ^{13}C NMR (100 MHz, $CDCl_3$) spectrum of compound **4**

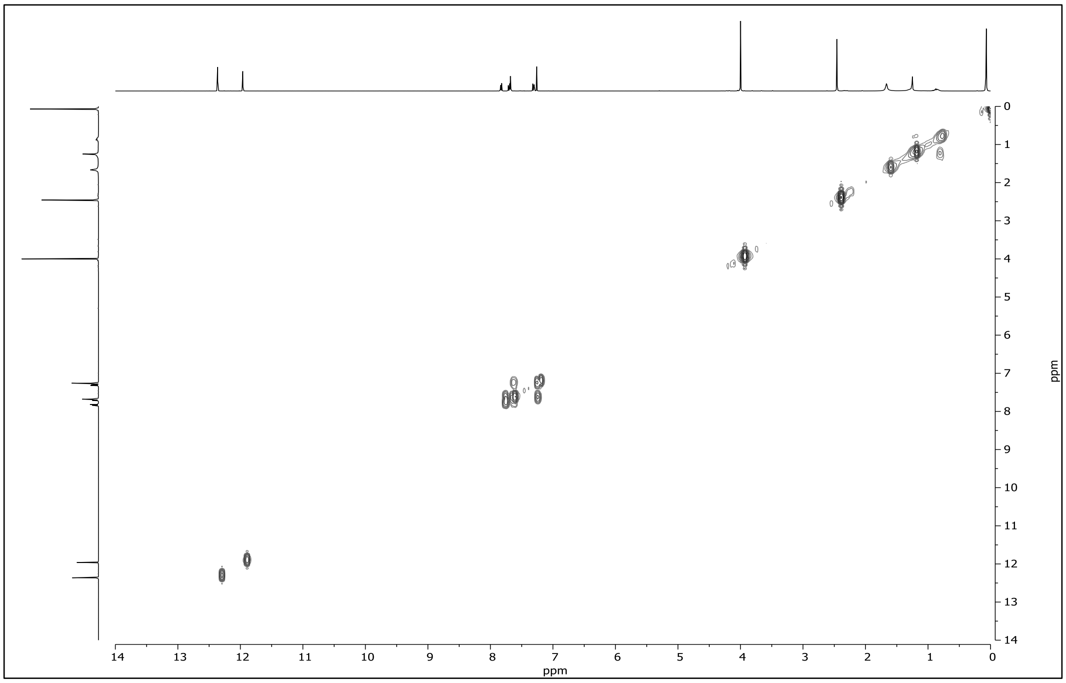

Figure S24. COSY ($CDCl_3$) spectrum of compound **4**

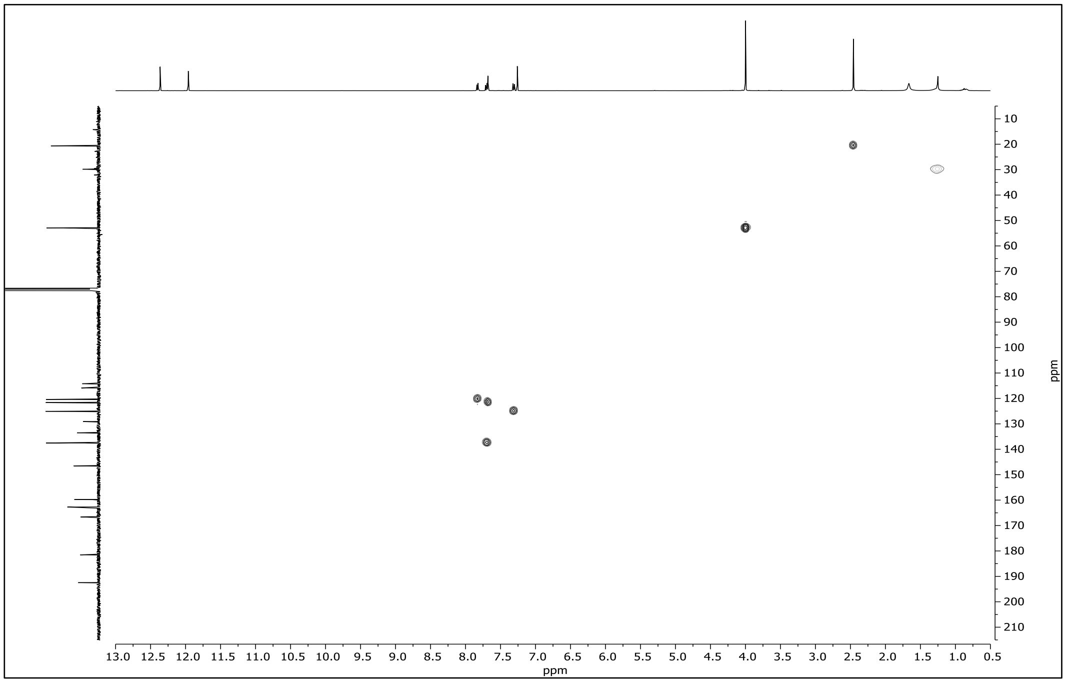

Figure S25. HSQC ($CDCl_3$) spectrum of compound **4**

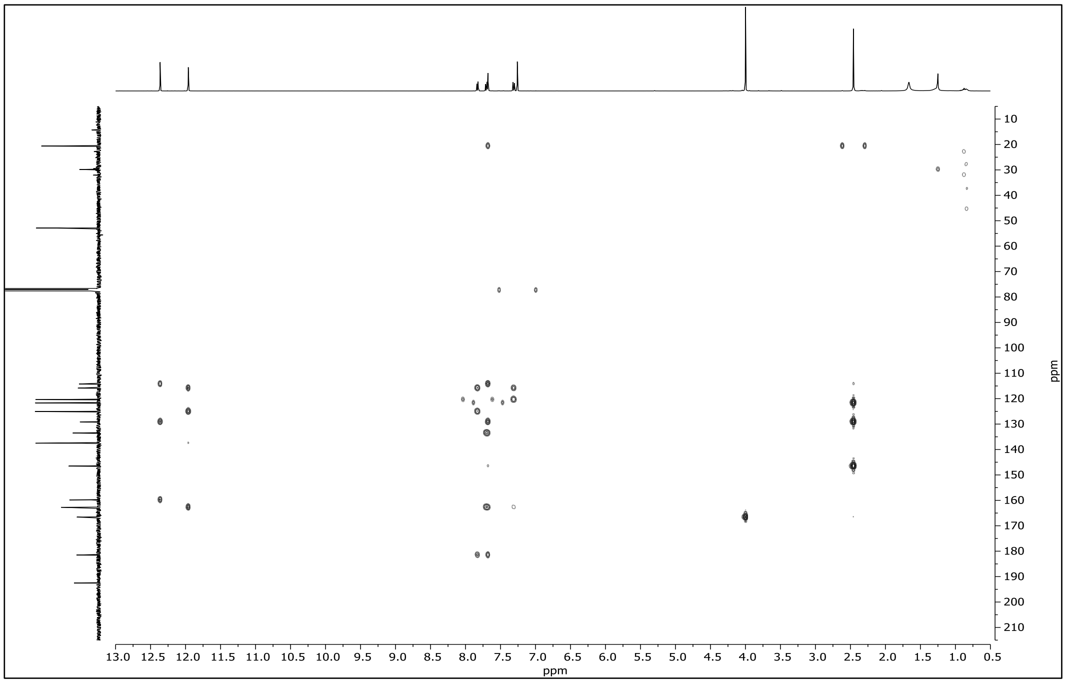

Figure S26. HMBC ($CDCl_3$) spectrum of compound **4**

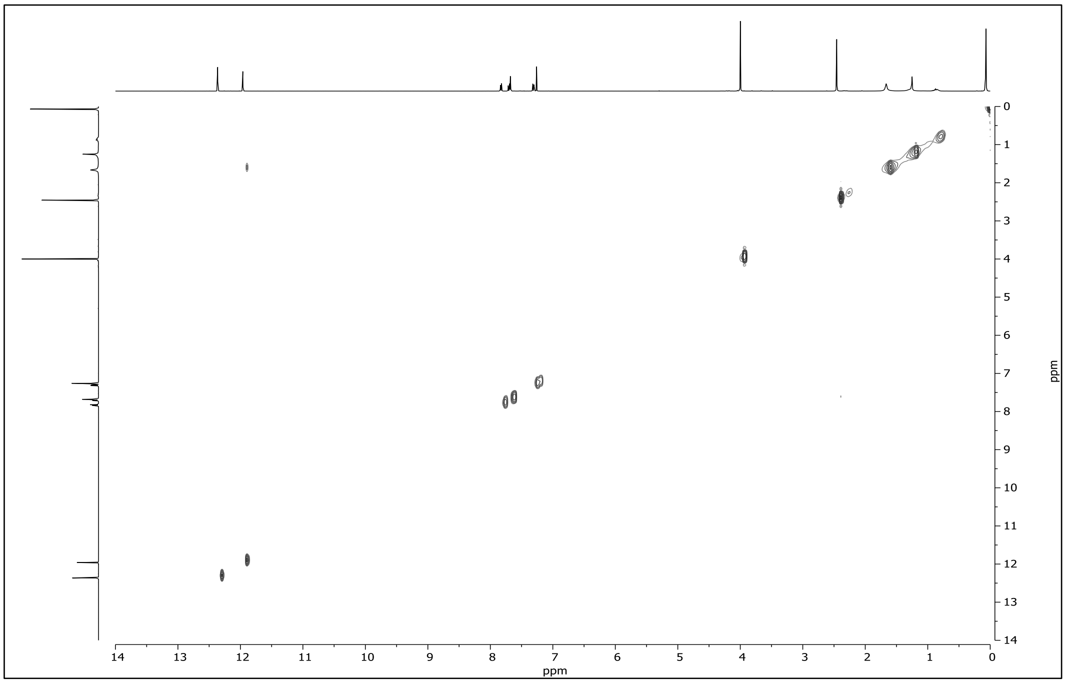

Figure S27. NOESY ($CDCl_3$) spectrum of compound **4**

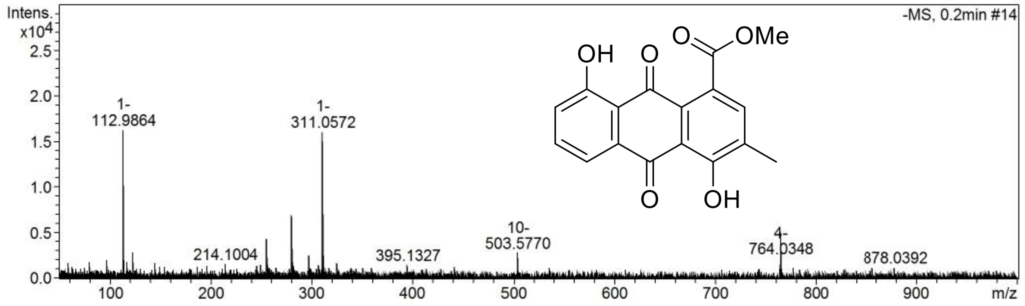

Meas. m/z	#	Ion Formula	m/z	|err| [mDa]	err [ppm]	Mean err [ppm]	rdb	N-Rule	e¯ Conf	mSigma
311.057214	1	C17H11O6	311.056112	1.1	3.5	-5.8	12.5	ok	even	14.9
	1	C11H16ClO8	311.053919	3.3	10.6	-13.7	3.5	ok	even	138.9

Figure S28. MS spectrum of compound **4**

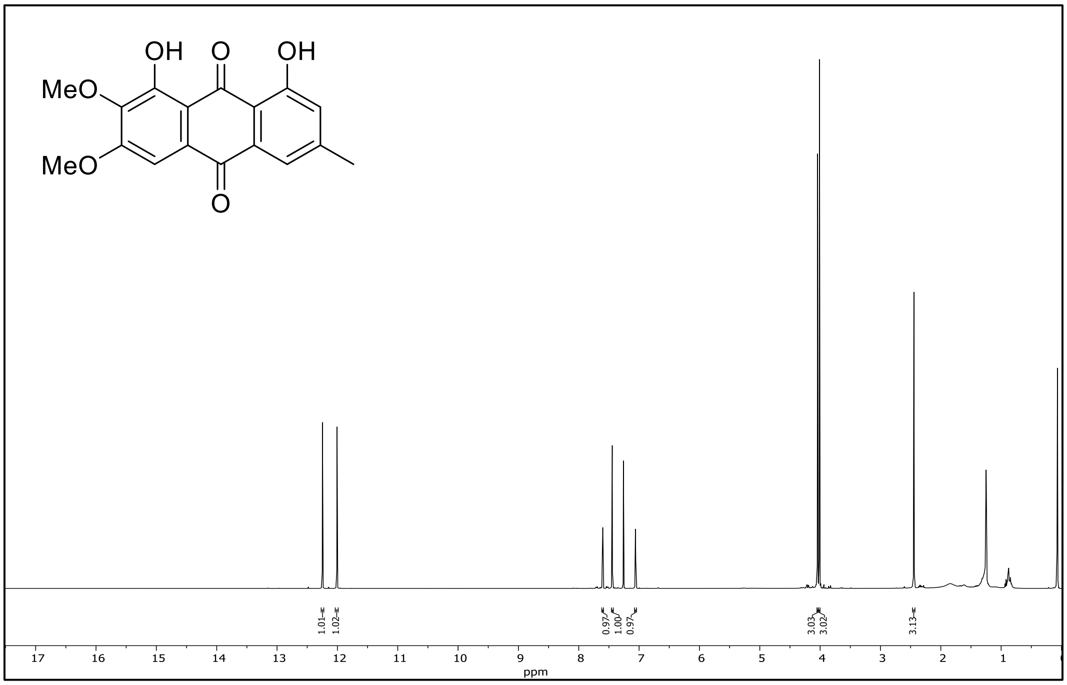

Figure S29. ^{1}H NMR (400 MHz, $CDCl_3$) spectrum of 7-Methoxyphyscion **5**

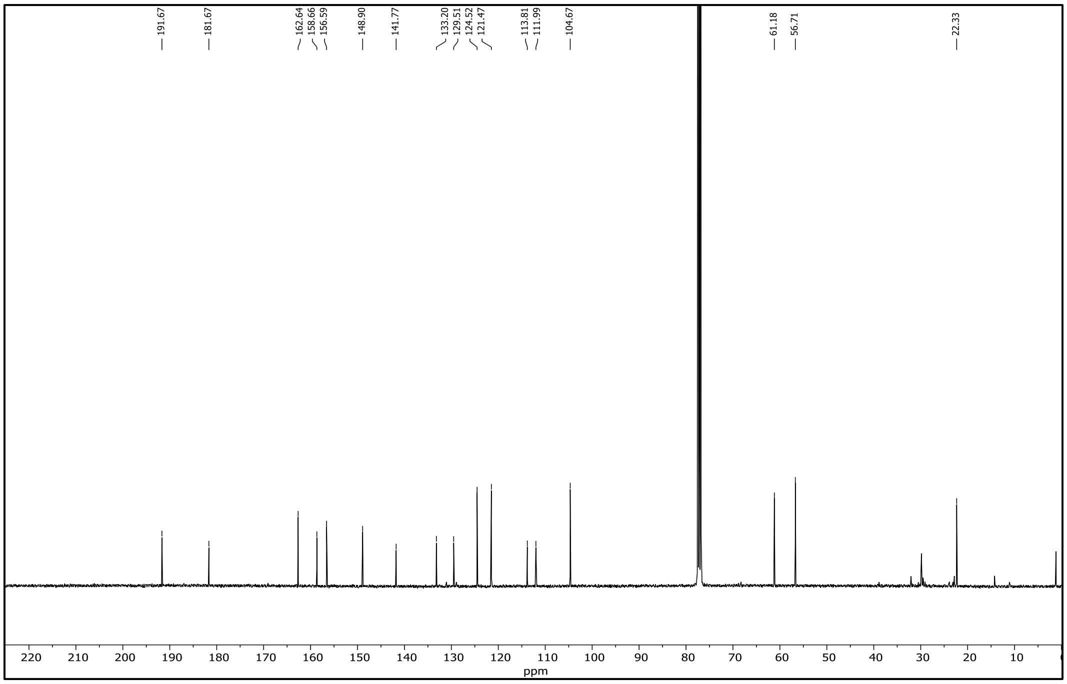

Figure S30. ^{13}C NMR (100 MHz, $CDCl_3$) spectrum of 7-Methoxyphyscion **5**

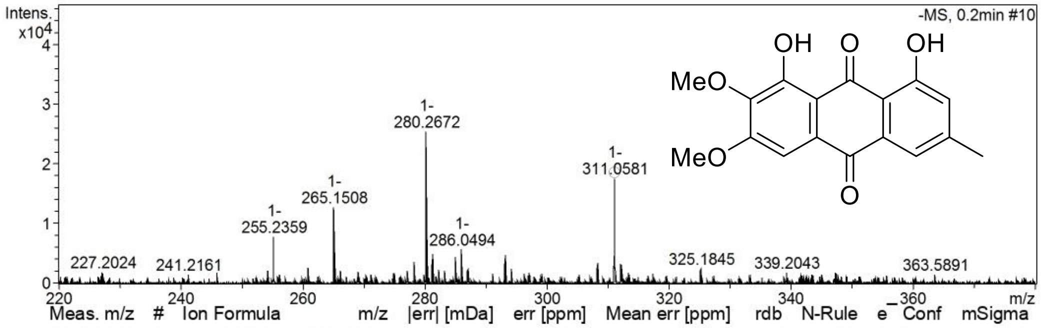

Meas. m/z	#	Ion Formula	m/z	|err| [mDa]	err [ppm]	Mean err [ppm]	rdb	N-Rule	e⁻ Conf	mSigma
311.058082	1	C17H11O6	311.056112	2.0	-6.3	-8.3	12.5	ok	even	4.6
	2	C14H15O8	311.077241	19.2	61.6	59.6	7.5	ok	even	21.1
	3	C21H11O3	311.071368	13.3	-42.7	40.8	16.5	ok	even	21.9
	4	C13H11O9	311.040856	17.2	55.4	-57.3	8.5	ok	even	27.2
	5	C24H7O	311.050238	7.8	25.2	-27.1	21.5	ok	even	39.8
	1	C15H16ClO5	311.069175	11.1	35.7	33.0	7.5	ok	even	140.3
	2	C18H12ClO3	311.048046	10.0	-32.3	-34.9	12.5	ok	even	141.3
	3	C11H16ClO8	311.053919	4.2	-13.4	-16.0	3.5	ok	even	142.3
	4	C22H12Cl	311.063302	5.2	-16.8	14.1	16.5	ok	even	145.4

Figure S31. MS spectrum of 7-Methoxyphyscion **5**

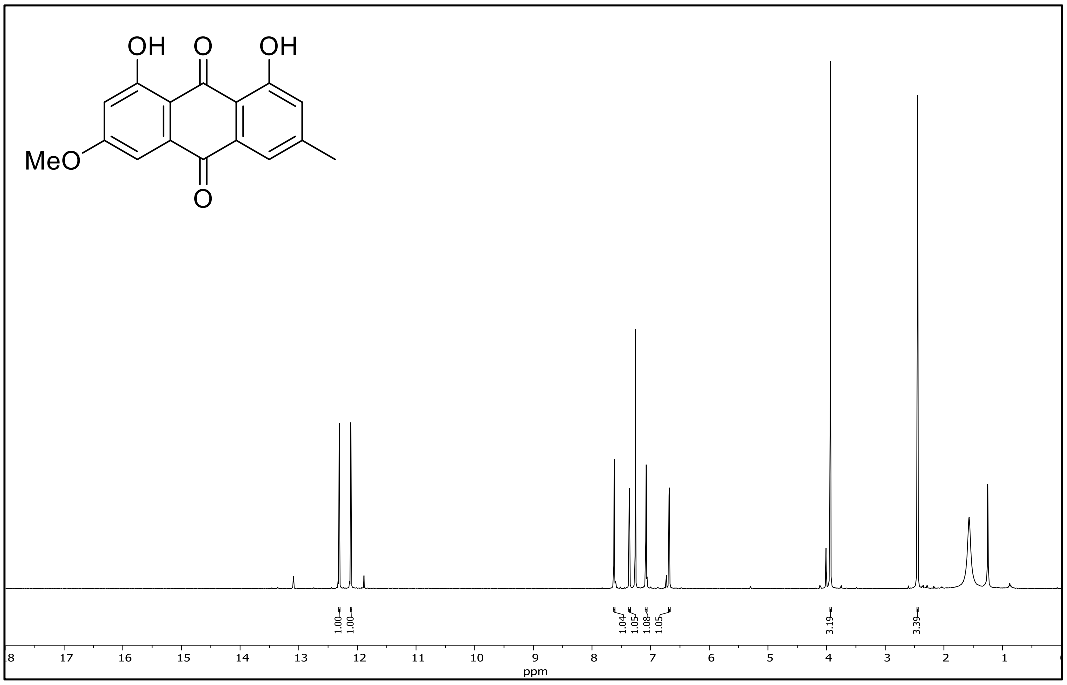

Figure S32 ^{1}H NMR (400 MHz, $CDCl_3$) spectrum of Physcion **6**

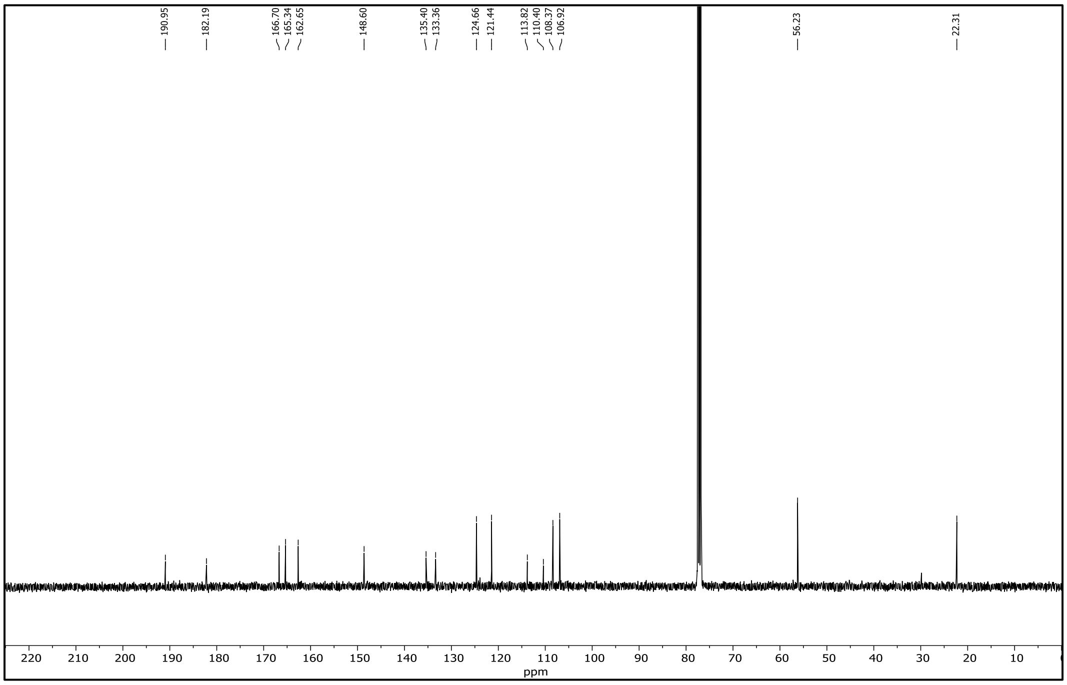

Figure S33 ^{13}C NMR (100 MHz, $CDCl_3$) spectrum of Physcion **6**

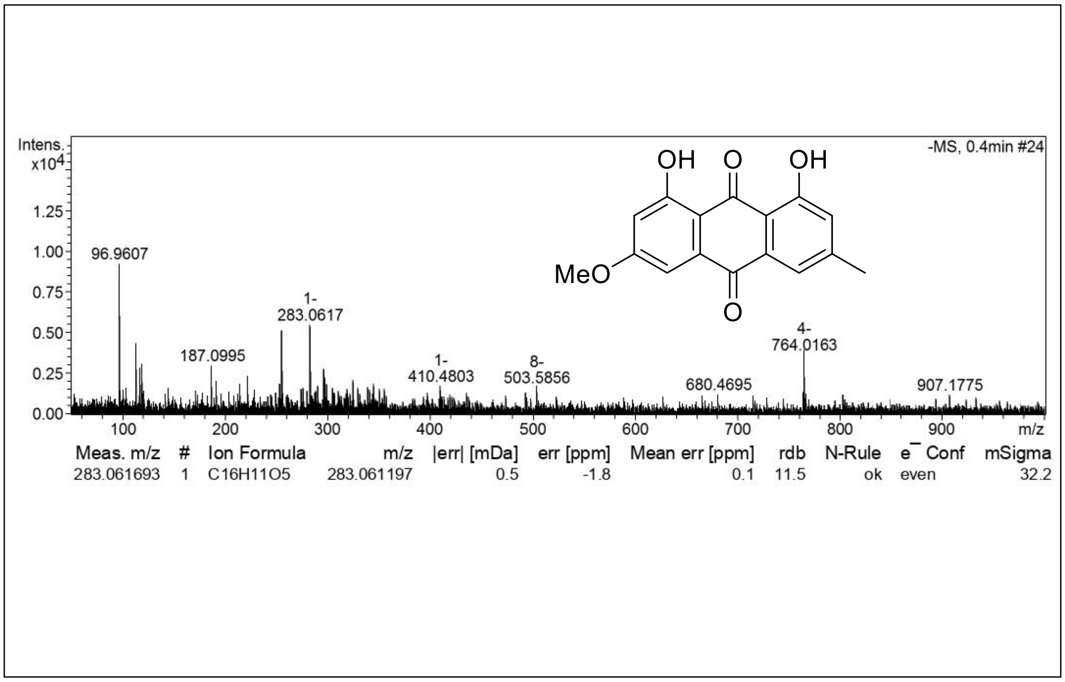

Figure S34. MS spectrum of Physcion **6**

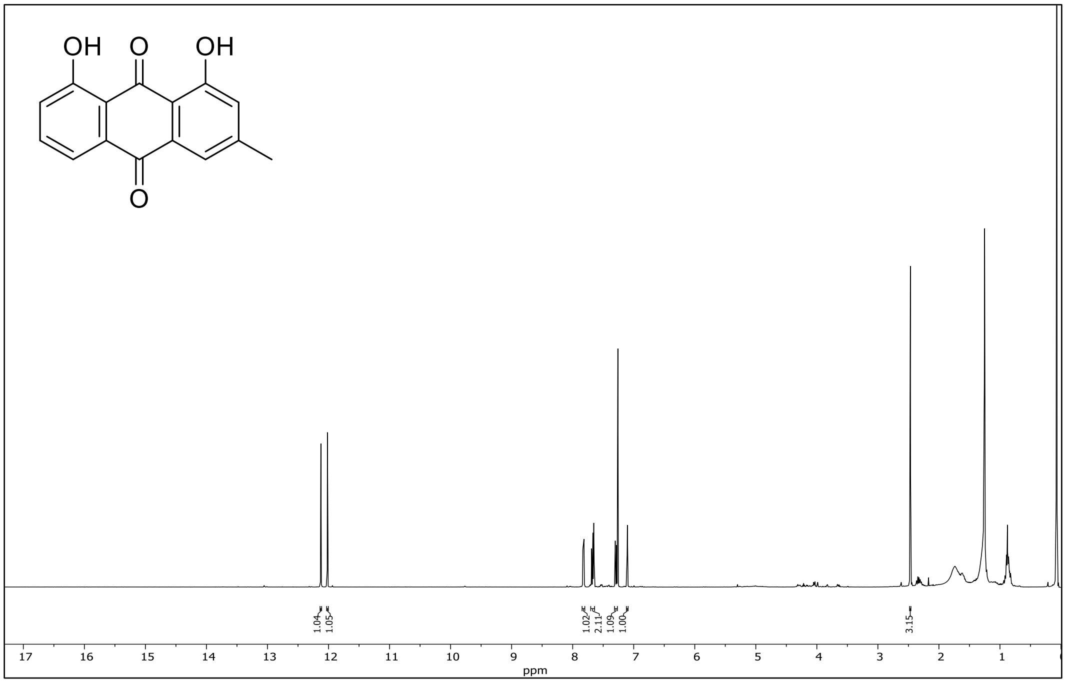

Figure S35. ^{1}H NMR (400 MHz, $CDCl_3$) spectrum of Chrysophanol **7**

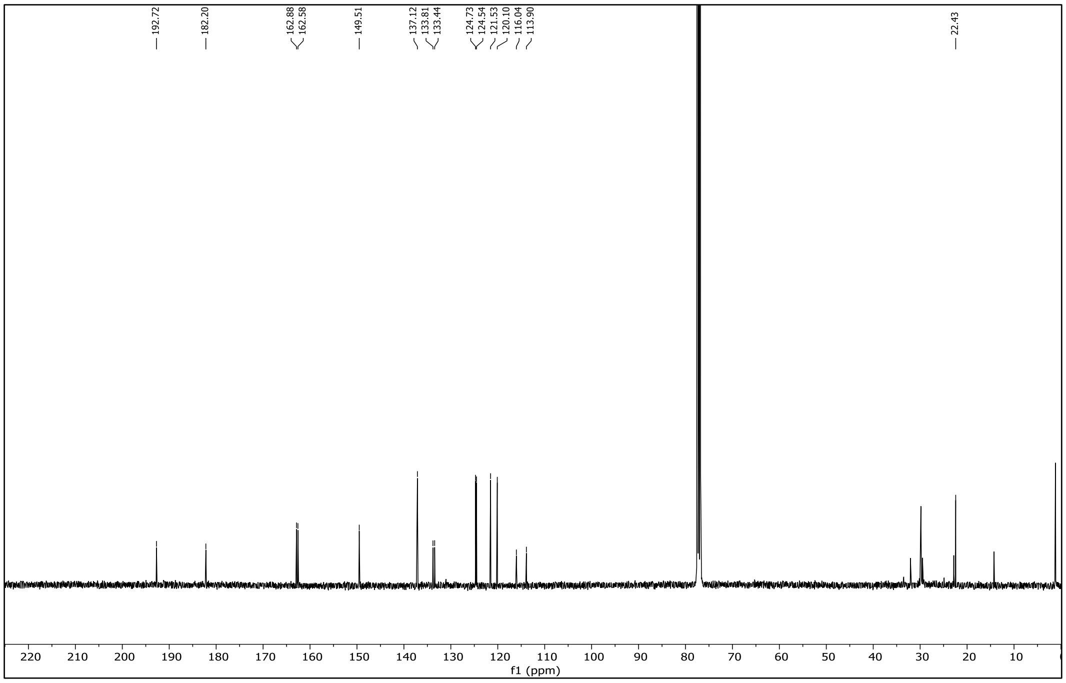

Figure S36. ^{13}C NMR (100 MHz, $CDCl_3$) spectrum of Chrysophanol **7**

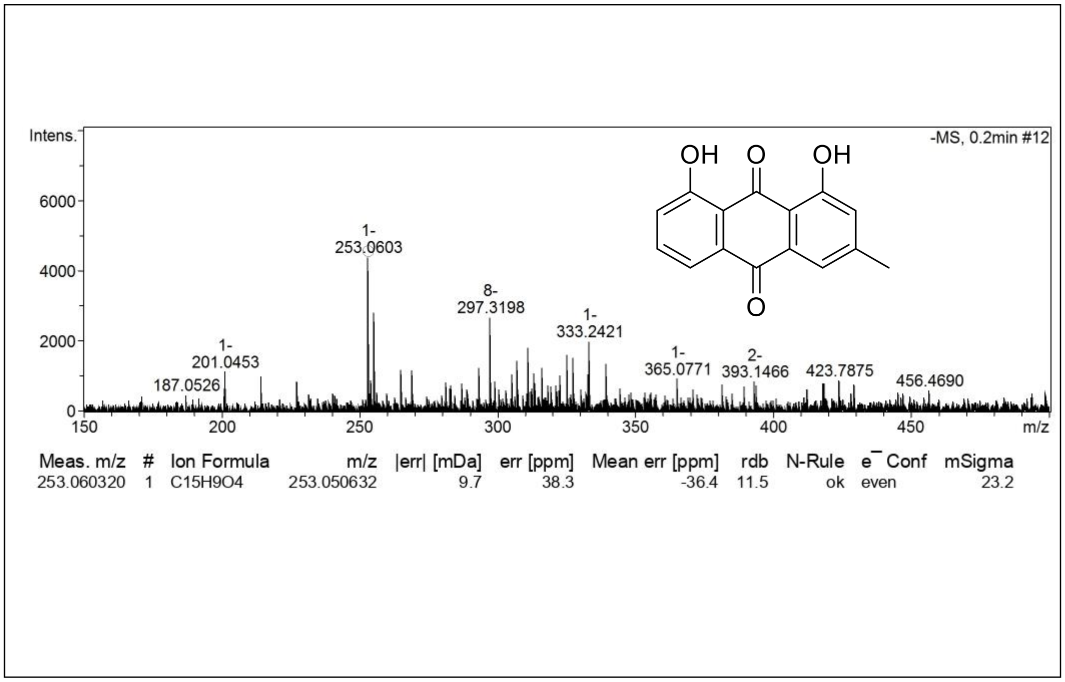

Figure S37. MS spectrum of Chrysophanol **7**

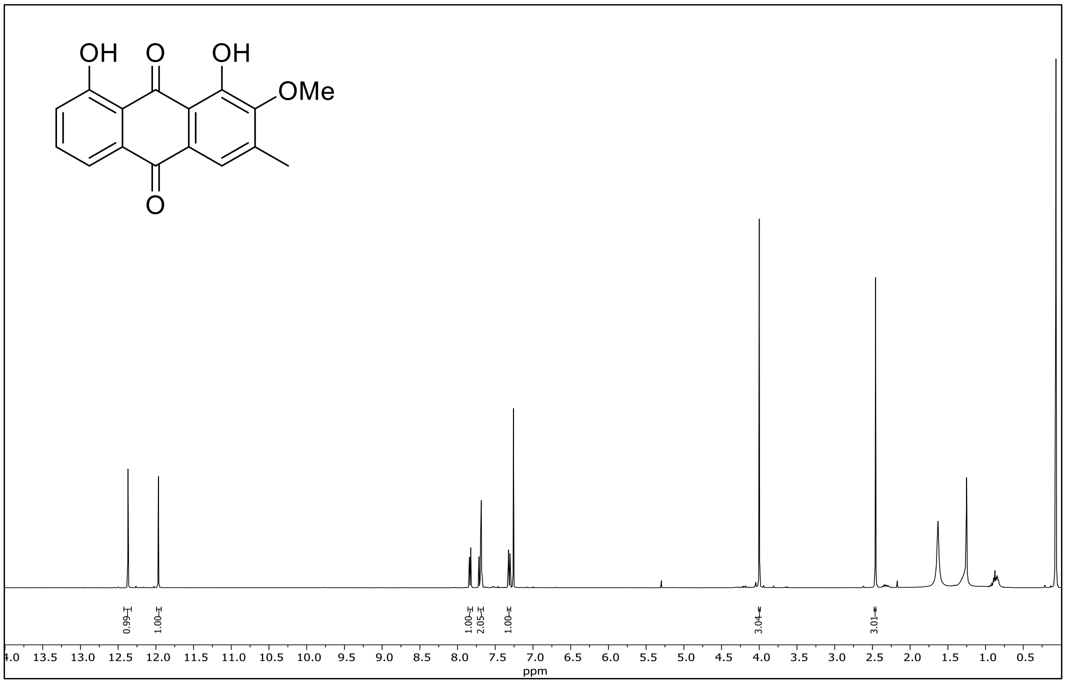

Figure S38. ^{1}H NMR (400 MHz, $CDCl_3$) spectrum of 2-Methoxychrysophanol **8**

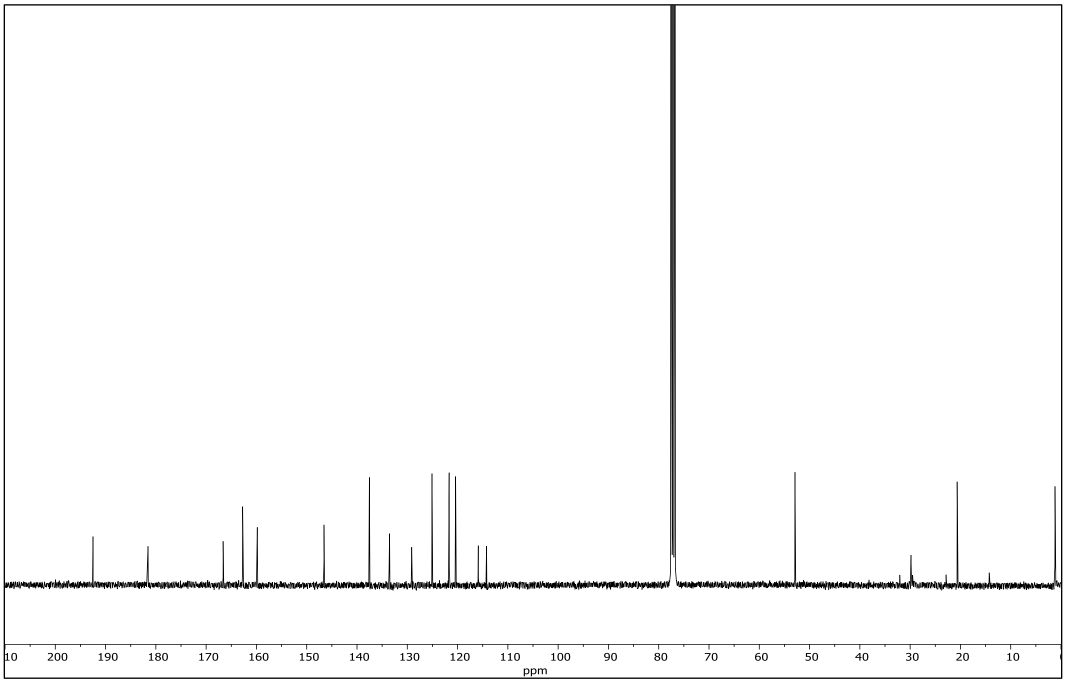

Figure S39. ^{13}C NMR (100 MHz, $CDCl_3$) spectrum of 2-Methoxychrysophanol **8**

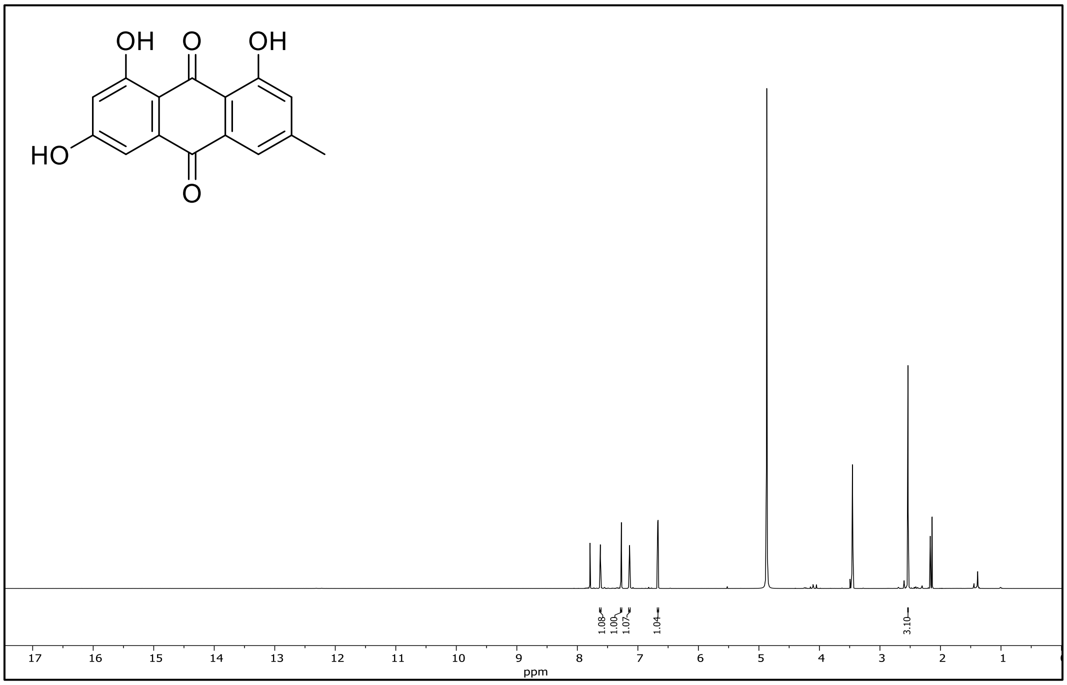

Figure S40. ^{1}H NMR (400 MHz, $CDCl_3$+CD_3OD) spectrum of Emodin **9**

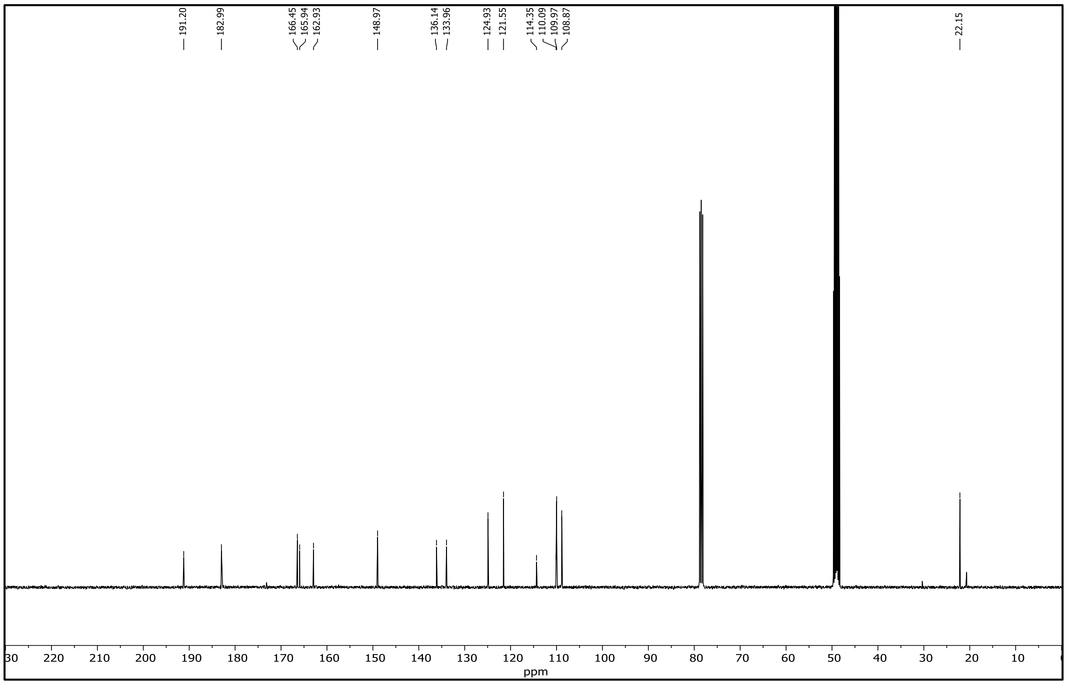

Figure S41. ^{13}C NMR (100 MHz, $CDCl_3$+CD_3OD) spectrum of Emodin **9**

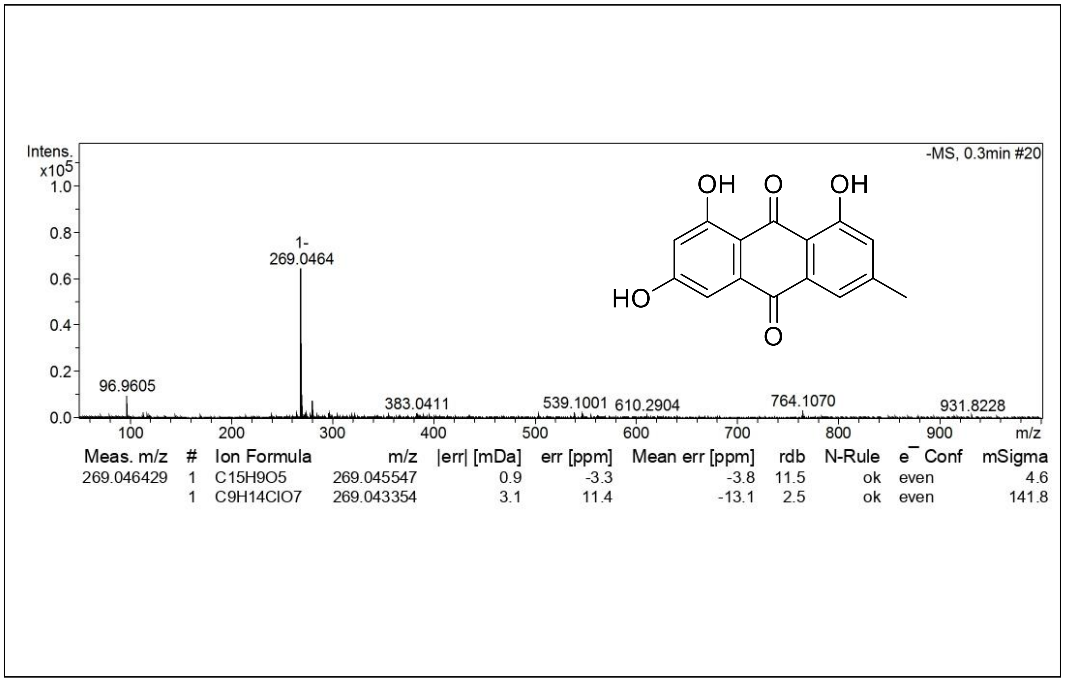

Figure S42. MS spectrum of Emodin **9**

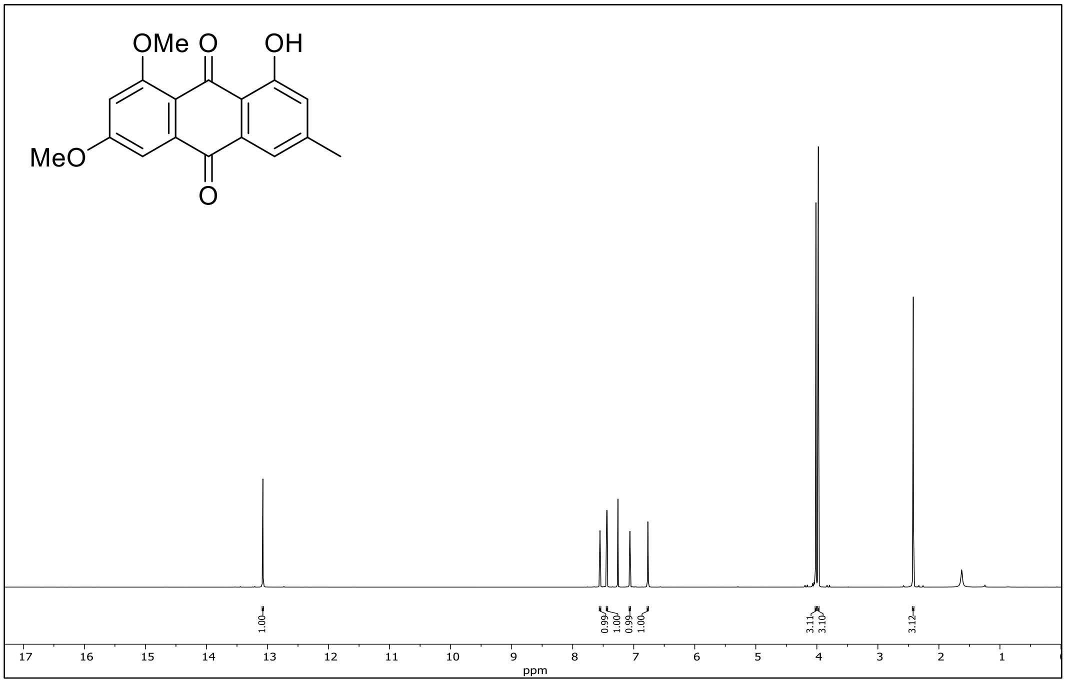

Figure S43. ^{1}H NMR (400 MHz, $CDCl_3$) spectrum of Emodin-6,8-dimethyl ether **10**

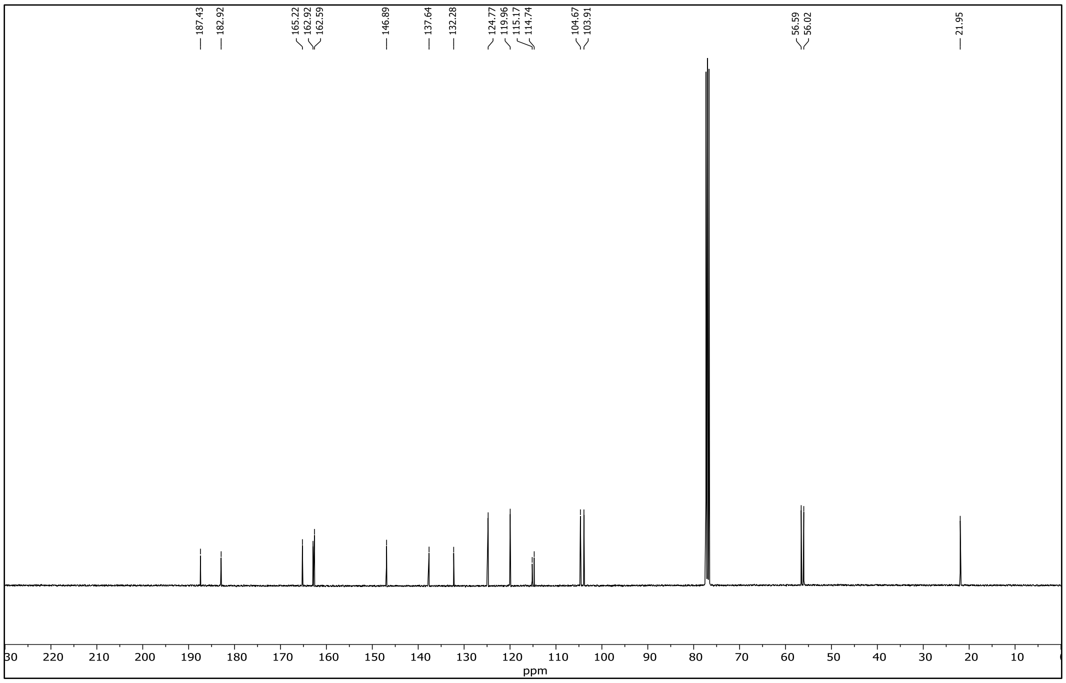

Figure S44. ^{13}C NMR (100 MHz, $CDCl_3$) spectrum of Emodin-6,8-dimethyl ether **10**

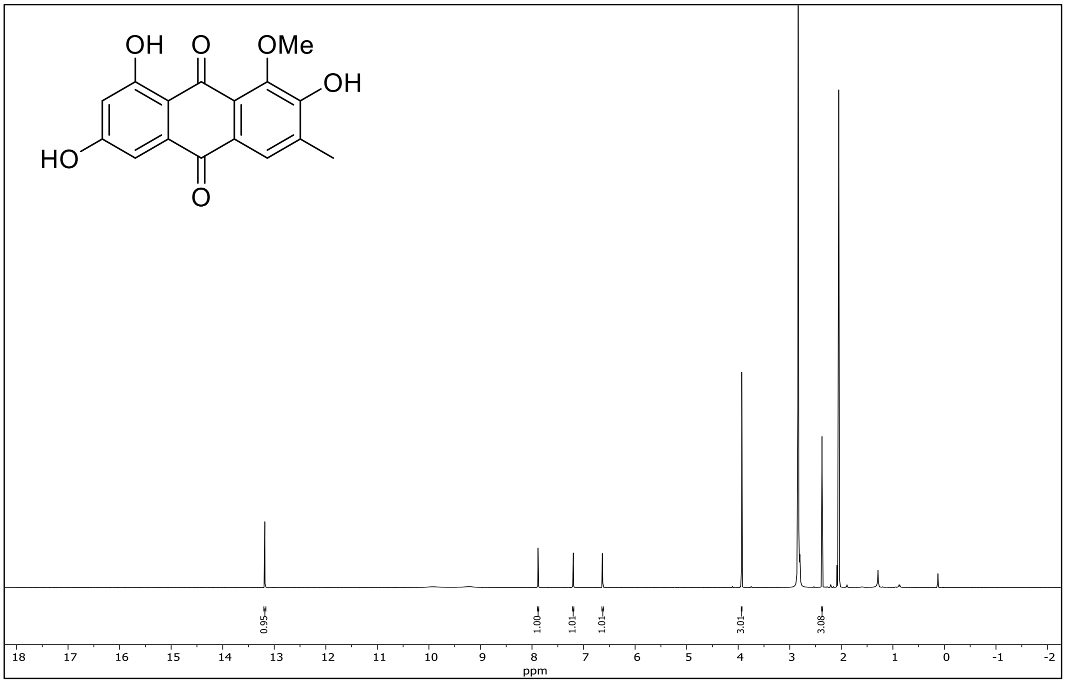

Figure S45. ^{1}H NMR (400 MHz, $CDCl_3$) spectrum of 2-Hydroxyemodin-1-methyl ether **11**

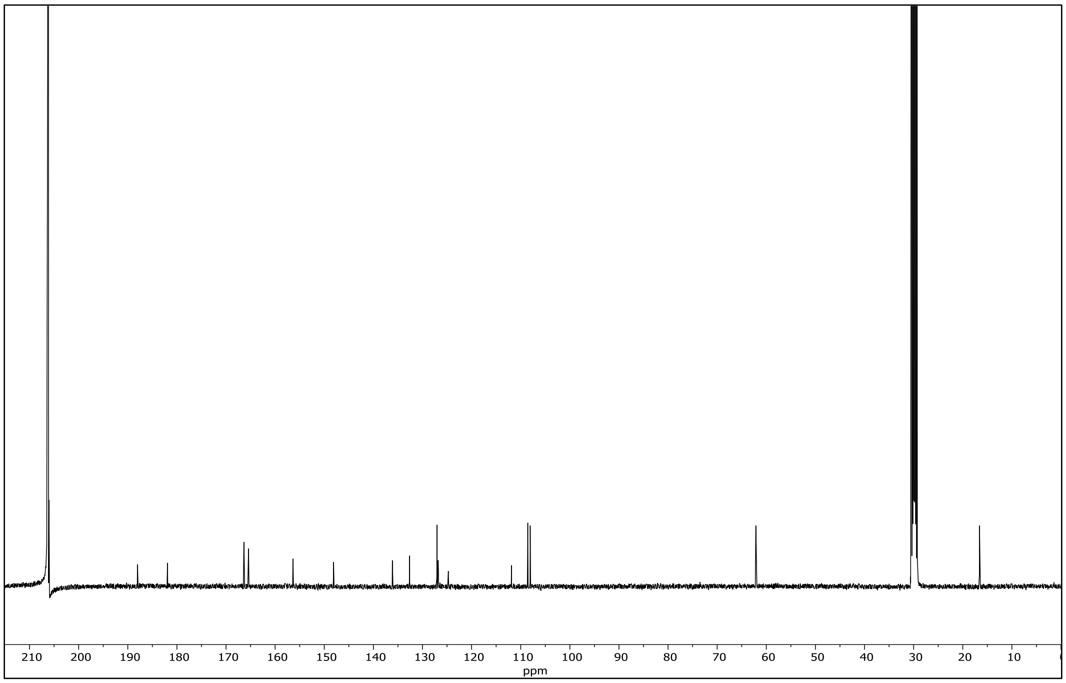

Figure S46. ^{13}C NMR (400 MHz, $CDCl_3$) spectrum of 2-Hydroxyemodin-1-methyl ether **11**

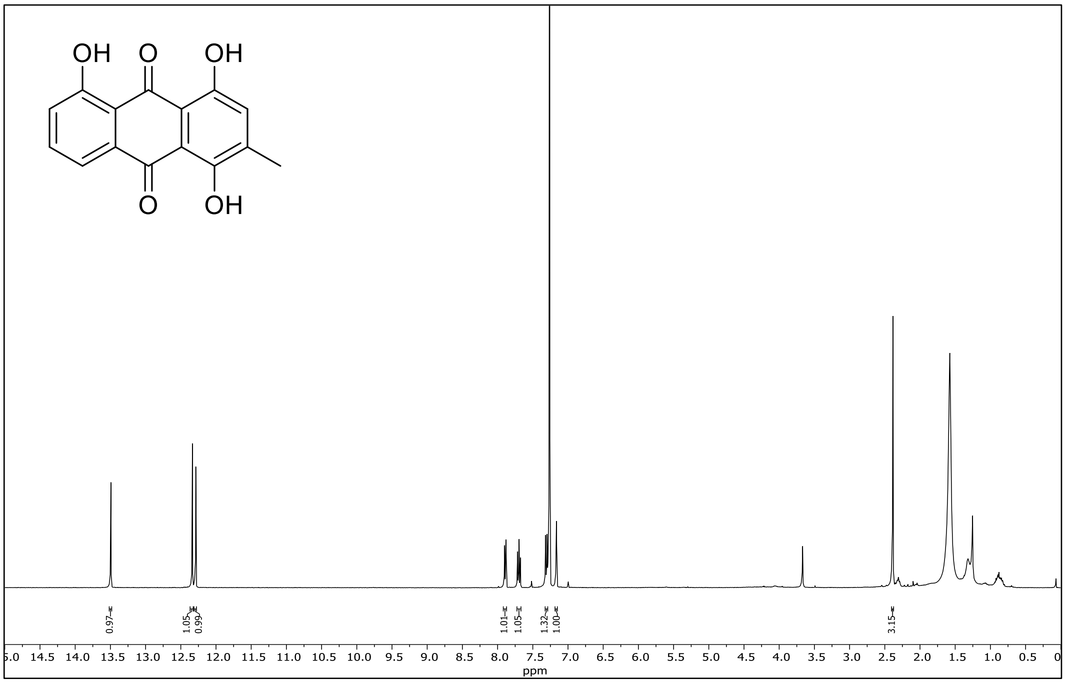

Figure S47. ^{1}H NMR (400 MHz, $CDCl_3$) spectrum of Islandicin **12**

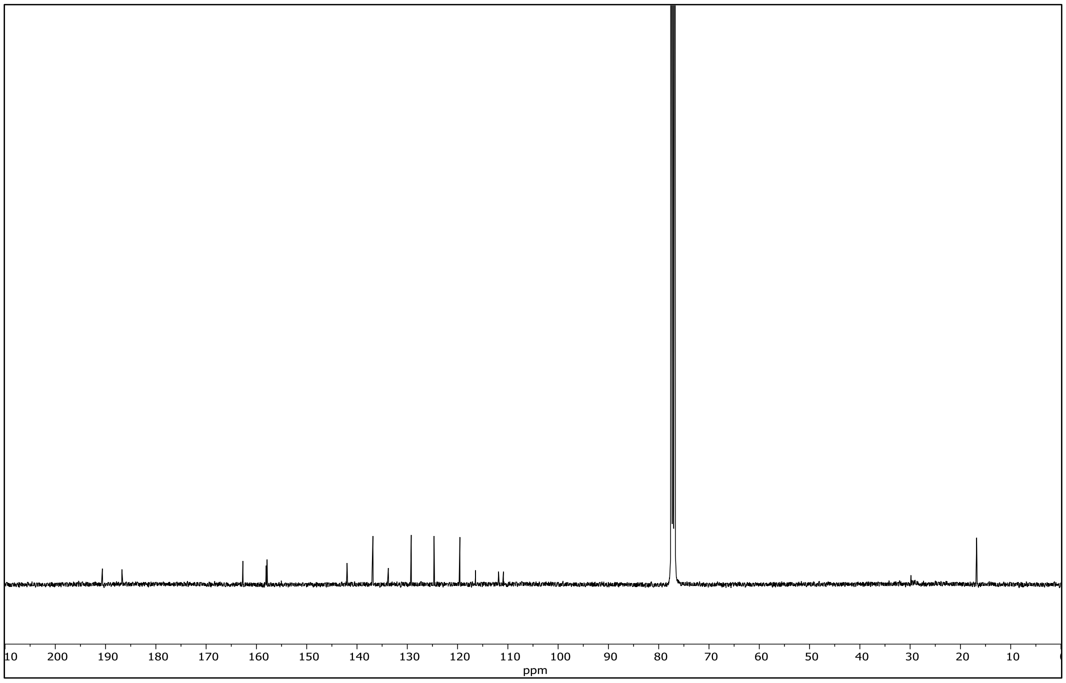

Figure S48. ^{13}C NMR (100 MHz, $CDCl_3$) spectrum of Islandicin **12**
